# Characterization of two family AA9 LPMOs from *Aspergillus tamarii* with distinct activities on xyloglucan reveals structural differences linked to cleavage specificity

**DOI:** 10.1371/journal.pone.0235642

**Published:** 2020-07-08

**Authors:** Antonielle V. Monclaro, Dejan M. Petrović, Gabriel S. C. Alves, Marcos M. C. Costa, Glaucia E. O. Midorikawa, Robert N. G. Miller, Edivaldo X. F. Filho, Vincent G. H. Eijsink, Anikó Várnai

**Affiliations:** 1 Laboratory of Enzymology, University of Brasília, Campus Universitário Darcy Ribeiro, Brasília, Brazil; 2 Faculty of Chemistry, Biotechnology and Food Science, Norwegian University of Life Sciences (NMBU), Ås, Norway; 3 Laboratory of Microbiology, University of Brasília, Campus Universitário Darcy Ribeiro, Brasília, Brazil; 4 Brazilian Agricultural Research Corporation, Embrapa CENARGEN, Brasília, Brazil; Institut National de la Recherche Agronomique, FRANCE

## Abstract

*Aspergillus tamarii* grows abundantly in naturally composting waste fibers of the textile industry and has a great potential in biomass decomposition. Amongst the key (hemi)cellulose-active enzymes in the secretomes of biomass-degrading fungi are the lytic polysaccharide monooxygenases (LPMOs). By catalyzing oxidative cleavage of glycoside bonds, LPMOs promote the activity of other lignocellulose-degrading enzymes. Here, we analyzed the catalytic potential of two of the seven AA9-type LPMOs that were detected in recently published transcriptome data for *A*. *tamarii*, namely *At*AA9A and *At*AA9B. Analysis of products generated from cellulose revealed that *At*AA9A is a C4-oxidizing enzyme, whereas *At*AA9B yielded a mixture of C1- and C4-oxidized products. *At*AA9A was also active on cellopentaose and cellohexaose. Both enzymes also cleaved the β-(1→4)-glucan backbone of tamarind xyloglucan, but with different cleavage patterns. *At*AA9A cleaved the xyloglucan backbone only next to unsubstituted glucosyl units, whereas *At*AA9B yielded product profiles indicating that it can cleave the xyloglucan backbone irrespective of substitutions. Building on these new results and on the expanding catalog of xyloglucan- and oligosaccharide-active AA9 LPMOs, we discuss possible structural properties that could underlie the observed functional differences. The results corroborate evidence that filamentous fungi have evolved AA9 LPMOs with distinct substrate specificities and regioselectivities, which likely have complementary functions during biomass degradation.

## Introduction

For many years, enzymatic conversion of plant polysaccharides was thought to be achieved exclusively by a consortium of hydrolytic enzymes, i.e. glycoside hydrolases (GHs) such as cellulases. In 2010, however, oxidative enzymes, today referred to as lytic polysaccharide monooxygenases (LPMOs), were also reported to contribute to the depolymerization of recalcitrant polysaccharides [1]. Since then, it has been shown that the action of these copper-dependent LPMOs decreases the recalcitrance of plant cell walls and boosts GH activity, thus enabling more efficient hydrolysis of biomass [2–7]. LPMOs are classified in the CAZy database [8] among the Auxiliary Activities (AAs), where they are currently grouped into families AA9, AA10, AA11, AA13, AA14, AA15, and AA16. LPMOs, in particular those belonging to the AA9 family, differ in terms of substrate specificity and regioselectivity. Next to cellulose, members of the AA9 family may cleave a variety of β-glucans [9, 10] as well as xylan [11, 12]. Some specifically oxidize the C1 or the C4 carbon, whereas others can oxidize both C1 and C4 carbons of the targeted β-(1→4)-glycosidic bond [2, 13, 14]. Among the AA9 LPMOs, several [9, 10, 15, 16] have been reported to be active on tamarind xyloglucan. This xyloglucan, a soluble polysaccharide, has a β-(1→4)-linked D-glucan backbone with a cellotetraose repeating unit where three out of four D-glucosyl units are substituted [17]. To date, two cleavage patterns have been reported for LPMOs regarding xyloglucan activity, which can be easily distinguished by mass spectrometric analysis. Some LPMOs yield a clustered product profile because they only cleave next to unsubstituted glucosyl units (e.g. *Nc*AA9C [9], here referred to as “substitution-intolerant” xyloglucan-active LPMOs), while others can cleave the xyloglucan backbone irrespective of substitutions and thus produce a myriad of products (e.g. *Gt*AA9A-2 and *Fg*AA9A [15, 16], here referred to as “substitution-tolerant” xyloglucan-active LPMOs).

Filamentous fungi are recognized for their great potential in biomass decomposition in nature. These characteristics are utilized within many fields of the biotechnological industry, e.g. in enzyme production, biodegradation of agro-industrial wastes, and a variety of industrial fermentation processes for upgrading low-value feedstocks [18–20]. Among the frequently employed species within the genus *Aspergillus*, *Aspergillus tamarii*, a saprophyte fungus, is recognized as an efficient protease [21, 22] and xylanase [23–26] producer. So far, the (putative) LPMOs of *A*. *tamarii* have not gained much attention. With regard to putative LPMOs from aspergilli, LPMO activity has been shown, to date, for five AA9s from *A*. *fumigatus* [27, 28], seven AA9s from *A*. *terreus* [27], two AA9s [29, 30] and one AA13 [31] from *A*. *nidulans*, an AA9 from *A*. *niger* [32], an AA11 [33] and AA13 [31] from *A*. *oryzae*, and an AA16 from *A*. *aculeatus* [34], whereas structural data are available for an AA9 (*Af*AA9B) from *A*. *fumigatus* [35] and an AA11 [33] and AA13 [31] from *A*. *oryzae*. So far, xyloglucan activity by these LPMOs has remained unexplored, except for one LPMO from *A*. *nidulans* (AN3046) for which xyloglucan activity has been proposed [29].

More than 90% of fungal genomes contain genes encoding LPMOs in families AA9-11, AA13-14 and/or AA16. Fungi tend to contain multiple genes encoding different members of the same LPMO family, and this is particularly true for AA9s, where gene numbers may reach more than 30 in some cases [36–38]. The functional characterization of sets of enzymes of a particular family from lignocellulolytic microorganisms is crucial for understanding their roles in the degradation of lignocellulosic biomass. In this context, this study was conducted to clone and characterize AA9 LPMOs from *A*. *tamarii*. We report here the properties of *At*AA9A and *At*AA9B, with different activities determined on cellulose and xyloglucan.

## Materials and methods

### Cloning and expression of *Aspergillus tamarii* LPMOs

Sequences were accessed from RNA-seq data for analysis of gene expression in *Aspergillus tamarii* strain BLU37 grown on steam-exploded sugarcane bagasse as carbon source [39]. In total, seven expressed AA9 genes were identified in the transcriptome datasets, with sequences of the predicted proteins listed in S1 Table in S1 Appendix. Sequences were analyzed for domains corresponding to carbohydrate-active enzymes or domains of unknown function using the dbCAN2 metaserver [40] and by multiple sequence alignment with similar domains in other proteins in the UniProt database using MUSCLE [41]. The genes encoding full-length *At*AA9A, *At*AA9B, and *At*AA9G, excluding introns but including the native signal peptide, were codon optimized for *Pichia pastoris* (GenScript, Piscataway, NJ, USA). The synthetic genes were inserted into the pPink-GAP vector as previously described [42]. To generate truncated proteins containing the catalytic domain only, gene fragments encoding the AA9-domains of *At*AA9A (*At*AA9A-N; 702 nucleotides, encoding 234 residues) and *At*AA9B (*At*AA9B-N; 732 nucleotides, encoding 244 residues) were PCR amplified from the pPINK-GAP-*At*AA9A and pPINK-GAP-*At*AA9B constructs, respectively. PCR products were ligated into the pPINK_GAP_TaCel5A vector [42] using restriction enzymes *Eco*RI and *Acc*65I and the In-Fusion HD cloning kit (Clontech Laboratories, Mountain View, CA). The expression vectors were transformed into *P*. *pastoris* PichiaPink™ cells (Invitrogen, Carlsbad, CA, USA) and transformants screened for protein production in BMGY medium as previously described [42].

For the production of *At*AA9A-N and *At*AA9B-N, transformants with greatest expression for each protein were grown in 25 mL of BMGY medium (containing 1% (v/v) glycerol) in 250-mL Erlenmeyer flasks at 29°C and 200 rpm for 16 h. These pre-cultures were subsequently used to inoculate 500 mL BMGY medium (containing 1% (v/v) glycerol) in 2-L Erlenmeyer flasks, then incubated at 29°C and 200 rpm for 48 h. After 24 h of incubation, the media were supplemented with 1% (v/v) glycerol. Cells were removed by centrifugation at 8,000 *g* for 15 min at 4°C. The supernatants were dialyzed against 20 mM Tris-HCl pH 8.0 until they reached a conductivity of 4 mS.cm^-1^, then concentrated to 50 mL using a VivaFlow 200 tangential crossflow concentrator (MWCO 10 kDa, Sartorius Stedim Biotech Gmbh, Germany).

### Purification and Cu(II) saturation

*At*AA9A-N and *At*AA9B-N were purified using a two-step purification protocol, starting with anion exchange chromatography followed by size exclusion chromatography. The concentrated broth after buffer exchange (see above) was loaded onto a 5-mL Q Sepharose FF column (GE Healthcare BioSciences AB, Sweden) equilibrated with 20 mM Tris-HCl pH 8.0. The bound proteins were eluted by applying a linear gradient from 0 to 0.5 M NaCl in the same buffer. Fractions were analyzed by SDS-PAGE and those containing *At*AA9A-N or *At*AA9B-N then pooled, dialyzed against 20 mM BisTris-HCl pH 6.0 and concentrated to 2 mL using Amicon Ultra centrifugal filters (MWCO 10 kDa, Merck Millipore, Carrigtwohill, Ireland). The concentrated samples were applied to a 120-mL Superdex 75 16/600 gel filtration column (GE Healthcare BioSciences AB) in 20 mM BisTris-HCl pH 6.0 supplemented with 150 mM NaCl. Protein purity was analyzed by SDS-PAGE and the fractions containing *At*AA9A-N or *At*AA9B-N were pooled and concentrated to 1 mL using Amicon Ultra centrifugal filters (MWCO 10 kDa, Merck Millipore), followed by sterilization by filtration through a 0.2-μm syringe filter. Protein concentrations were determined by measuring absorbance at 280 nm, using theoretical extinction coefficients calculated with the ExPASy server [43] (*At*AA9A-N, 39545 M^-1^cm^-1^; *At*AA9B-N, 41620 M^-1^cm^-1^). *At*AA9A-N and *At*AA9B-N were saturated with Cu(II) by incubating the enzymes with an excess of CuSO_4_ (3:1 molar ratio of copper to enzyme) for 30 min at room temperature, as described previously [44]. The solution was then loaded onto a PD MidiTrap G-25 desalting column (GE Healthcare, UK), equilibrated with 20 mM BisTris-HCl pH 6.0. Fractions containing *At*AA9A-N and *At*AA9B-N, eluted with 1 mL of the same buffer, were collected and stored at 4°C before further use.

### *In silico* analysis of *A*. *tamarii* LPMOs

For phylogenetic analysis, a multiple sequence alignment of AA9 domains (without the C-terminal extension) was generated using MUSCLE [41] and the phylogenetic tree was generated using Interactive Tree of Life (iTOL) [45]. The sequence alignment of xyloglucan-active LPMOs was generated using T-Coffee’s Expresso tool [46]. Structural models of xyloglucan-active LPMOs were generated with SWISS-MODEL [47], using the templates with PDB IDs 3ZUD (for *At*AA9B-N and *Fg*AA9A-N), 4B5Q (for *Mc*AA9H), 4D7U (for MYCTH_79765 and *Pa*AA9H-N), 4EIR (for MYCTH_100518), 4EIS (for *Gt*AA9A-2-N, *Gt*AA9B, and *Mc*AA9B), 4QI8 (for MYCTH_85556), 5N05 (for *At*AA9A-N) and 6H1Z (for *Mc*AA9A-N and *Mc*AA9F). The images of protein structures were generated using PyMOL version 1.3 Schrödinger, LLC (New York, NY, USA).

### Substrates

The following substrates were employed for exploring the activity of *At*AA9A-N and *At*AA9B-N: phosphoric acid swollen cellulose (PASC) prepared from Avicel PH-101 (Sigma Aldrich, St. Louis, MO, USA), as described by Wood [48]; tamarind xyloglucan, cellohexaose (Glc_6_), cellopentaose (Glc_5_), Icelandic moss lichenan, and ivory nut mannan, all obtained from Megazyme International Ireland (Wicklow, Ireland); birchwood xylan from Sigma-Aldrich.

### Enzyme reactions

Reaction mixtures, in 200 μL total volumes, contained 0.2% (w/v) PASC or 1% (w/v) of the other substrates, and 1 μM of *At*AA9A-N or *At*AA9B-N in 50 mM of BisTris-HCl pH 6.0, supplied with 1 mM ascorbic acid as indicated. Purified recombinant cellobiose dehydrogenase (*Mt*CDH) from *Myriococcum thermophilum* [49], at a concentration of 1 μM, was also used as an electron donor in reactions with PASC, instead of ascorbic acid. Samples were incubated at 37°C with shaking at 1000 rpm for 18 h. After incubation, soluble and insoluble fractions were separated using a 96-well filter plate (Merck Millipore) and a Merck Millipore vacuum manifold. C4-oxidized cello-oligosaccharide standards were produced by incubating PASC with 1 μM *Nc*AA9C [50], using the same conditions as for *At*AA9A-N and *At*AA9B-N. C1-oxidized standards were generated in the same manner, using 1 μM *Nc*AA9F [51]. Product formation was analyzed by high-performance anion-exchange chromatography (HPAEC) and MALDI-TOF mass spectrometry (MS), as described below.

### Time course analysis and quantification of released oxidized products

Reaction mixtures with 0.2% (w/v) PASC, 1 μM of *At*AA9 and 1 mM ascorbic acid were set up in 800 μL total volumes in 50 mM of BisTris-HCl pH 6.0 and incubated as specified above. Samples (150 μl) were collected after 20, 40, 60, 120, and 240 min of incubation and boiled at 97°C for 10 min to stop the reaction. Soluble and insoluble fractions were separated using a 96-well filter plate (Merck Millipore) and a Merck Millipore vacuum manifold. Next, 25 μl of the soluble fractions were supplemented with 1 μL *Tr*Cel7A in 150 mM Na-acetate pH 4.75 (to a final concentration of 1 μM), followed by incubation at 37°C for 18 h in order to convert the solubilized oxidized oligosaccharides to the corresponding oxidized dimers. After the incubation, the samples were incubated at 97°C for 10 min to stop the reaction. For product quantification, cellobionic acid (as C1-oxidized) and C4-oxidized dimer standards were prepared as described before [5, 52].

### Analysis of enzyme products

Native and oxidized oligosaccharides were analyzed by HPAEC using a Dionex ICS-5000 system equipped with pulsed-amperometric detection (PAD) and a CarboPac PA1 analytical column with a CarboPac PA1 guard column (Dionex, Sunnyvale, CA, US). A 0.25 mL/min flow and 50-min gradient were employed as previously described [53]. Additional product analysis was performed by MALDI-TOF MS, using an Ultraflex MALDI-TOF/TOF instrument (Bruker Daltonics, Bremen, Germany) equipped with a nitrogen 337-nm laser beam, as described previously [1]. Prior to MALDI-TOF MS analysis, samples (1 μL) were spotted on an MTP 384 ground steel target plate TF (Bruker Daltonics) together with 1 μL of a saturated 2,5-dihydroxybenzoic acid solution and dried.

## Results

### Amino acid sequence analysis of *A*. *tamarii* AA9s

The genome of *A*. *tamarii* CBS 117626 has only recently been published, and it contains nine predicted proteins annotated as AA9 LPMOs [54]. Previous analysis of the transcriptome of *A*. *tamarii* BLU37 during cultivation on steam-exploded sugarcane bagasse as exclusive lignocellulosic carbon source [39] revealed seven expressed genes encoding putative AA9 enzymes, which we named *At*AA9A, *At*AA9B, *At*AA9C, *At*AA9D, *At*AA9E, *At*AA9F, and *At*AA9G (see S1 Table for the predicted sequences, S2 Table for related LPMOs, including AA9s found in the *A*. *tamarii* CBS 117626 genome, as well as the closest related characterized LPMOs from aspergilli, and S3 Table for predicted properties in S1 Appendix). All seven AA9 LPMOs are secreted, as predicted using the SignalP program [55]. Of these, *At*AA9D is a fragment only, *At*AA9E is a single-domain LPMO, *At*AA9A and *At*AA9G carry a C-terminal CBM1, whereas *At*AA9B, *At*AA9C, and *At*AA9F carry a 129-, 78- and 61-amino acid extension, respectively, at the C-terminal end, none of which are similar to any previously described domain (S1 and S3 Tables and S1 Fig in S1 Appendix). The C-terminal extension of *At*AA9F seems to be a region of low complexity, while *At*AA9B and *At*AA9C are likely to carry small C-terminal domains of unknown function (S3 Table in S1 Appendix). Blasting the C-terminus of *At*AA9B against the UniProt database (E = 0.001) resulted in 98 hits, all of which were LPMO sequences, with 96 originating from *Aspergillus* and *Penicillium* species (S1A Fig in S1 Appendix). This C-terminus potentially encodes a novel carbohydrate-binding module (CBM) that is characteristic to these species, with the sequence features are highlighted below in the Discussion below. Analysis of the C-terminus of *At*AA9C against the UniProt database and the recently published *A*. *tamarii* genome [54] revealed that it may be a truncated version of a domain of unknown function. Comparison of the C-terminus of *At*AA9C with the C-termini of proteins sharing >90% identity, which were all LPMOs from *Aspergillus* species, indicated that, in the *At*AA9C sequence derived from the RNA-seq data, this domain lacks ca. 50 amino acids (S1B Fig in S1 Appendix).

Multiple sequence alignment of the AA9 domains with the AA9 LPMOs characterized to date revealed that the closest characterized relative of *At*AA9A is *Ls*AA9A, a C4-oxidizing LPMO from *Lentinus similis* (UniProt ID, A0A0S2GKZ1; 58% sequence identity) [56], while the closest relative of *At*AA9B is *Ta*AA9A, a well-studied C1/C4-oxidizing LPMO from *Thermoascus aurantiacus* (UniProt ID, G3XAP7; 71% and 69% sequence similarity, respectively) [2, 57] (S2 Fig and S2 Table in S1 Appendix). The closest characterized relatives of the other predicted LPMOs are listed in S2 Table in S1 Appendix. While sequence similarities may be indicative of regioselectivity and substrate specificity, they are not 100% predictive (S2 Fig in S1 Appendix and as discussed below).

### Heterologous expression of *A*. *tamarii* AA9s

Of the seven AA9 LPMOs identified in the transcriptome of *A*. *tamarii* BLU37, five were upregulated after 48 hours when growing *A*. *tamarii* on sugarcane bagasse [39] (S2 Table in S1 Appendix), indicating a role in lignocellulosic biomass degradation. As a first step to understanding the LPMO potential of *A*. *tamarii*, we attempted to clone three of the five upregulated LPMOs, namely *At*AA9A, *At*AA9B and *At*AA9G, with and without the C-terminal extension after the AA9 domains. *At*AA9D was omitted because the sequence was incomplete, and *At*AA9E was omitted because of sequence ambiguities. We successfully expressed in *P*. *pastoris* the catalytic domains of two of the three other LPMOs, namely *At*AA9A-N and *At*AA9B-N. The catalytic domain of *At*AA9G was also expressed but in low quantities so we decided to focus on *At*AA9A-N and *At*AA9B-N. *At*AA9A-N and *At*AA9B-N were purified to homogeneity using two chromatographic steps (S3 Fig in S1 Appendix), and further characterized.

Electrophoretic analysis revealed that recombinant *At*AA9A-N and *At*AA9B-N had a slightly higher apparent molecular mass (27 kDa and 29 kDa, respectively) than the theoretical values (23 kDa and 24 kDa, respectively). This modest difference could be due to low levels of glycosylation. *At*AA9A-N is predicted to have three potential *O*-glycosylation sites (Ser29, Thr37, and Thr42) and *At*AA9B-N is predicted to have one potential *N*- (Asn135) and one potential *O*-glycosylation site (Ser34), as predicted by the NetNGlyc v1.0 [58] and NetOGlyc v4.0 [59] servers of the Technical University of Denmark. Based on the position of these amino acids in the predicted structure (models built with *Ls*AA9A [PDB:5ACI] and *Ta*AA9A [PDB:2YET], respectively; more details below), only Ser29 in *At*AA9A is close to the catalytic surface, but still at a distance where an effect of a possible glycosylation on LPMO activity is unlikely.

### Cellulolytic activity of *At*AA9A-N and *At*AA9B-N

**Fig 1:**
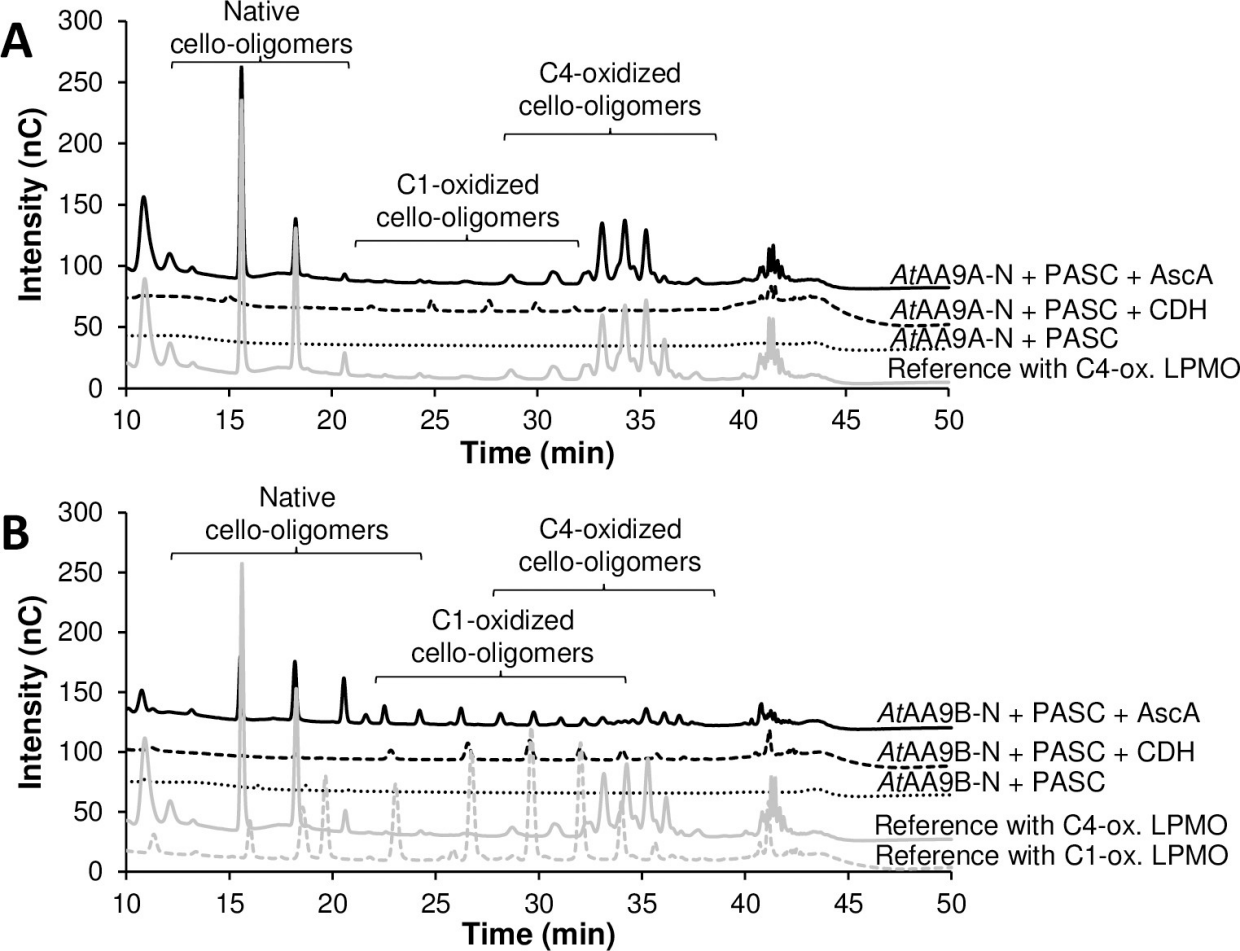
Oxidative cleavage of PASC by (A) *At*AA9A-N and (B) *At*AA9B-N. The graphs are HPAEC-PAD profiles of reaction mixtures containing 1 μM LPMO and 0.2% (w/v) PASC in 50 mM of BisTris-HCl pH 6.0, without electron donor (dotted black lines), with 1 mM ascorbic acid (AscA) as electron donor (solid black lines) or with 1 μM *Mt*CDH as electron donor (dashed black lines), after incubation at 37°C for 18 h. For identification of C1- and C4-oxidized cello-oligosaccharides, product mixtures generated in reactions with strictly C1-oxidizing 1 μM *Nc*AA9F (dashed grey lines) or strictly C4-oxidizing *Nc*AA9C (solid grey lines), respectively, were used as a reference.

The recombinant *At*AA9A-N and *At*AA9B-N were active on phosphoric acid-swollen cellulose (PASC), but with different regioselectivities (Fig 1 and S4 Fig in S1 Appendix). *At*AA9A-N generated native and C4-oxidized cello-oligosaccharides only (Fig 1A), whilst *At*AA9B-N generated both C1- and C4-oxidized (as well as native) cello-oligosaccharides (Fig 1B). No products were detected in control reactions without electron donor. When using *Mt*CDH as an electron donor, *Mt*CDH oxidized the reducing end of all solubilized cello-oligosaccharides, hence no native cello-oligosaccharides or C4-oxidized cello-oligosaccharides (or on-column degradation products thereof [60]) were detected, whereas small amounts of C1-oxidized cello-oligosaccharides were detected for both LPMOs (Fig 1A and 1B). These C1-oxidized cello-oligosaccharides, which were not observed in the control reaction without CDH and which must derive from native products generated by the LPMO, shows that CDH indeed was capable of driving the reactions with both LPMOs (Fig 1A and 1B).

**Fig 2:**
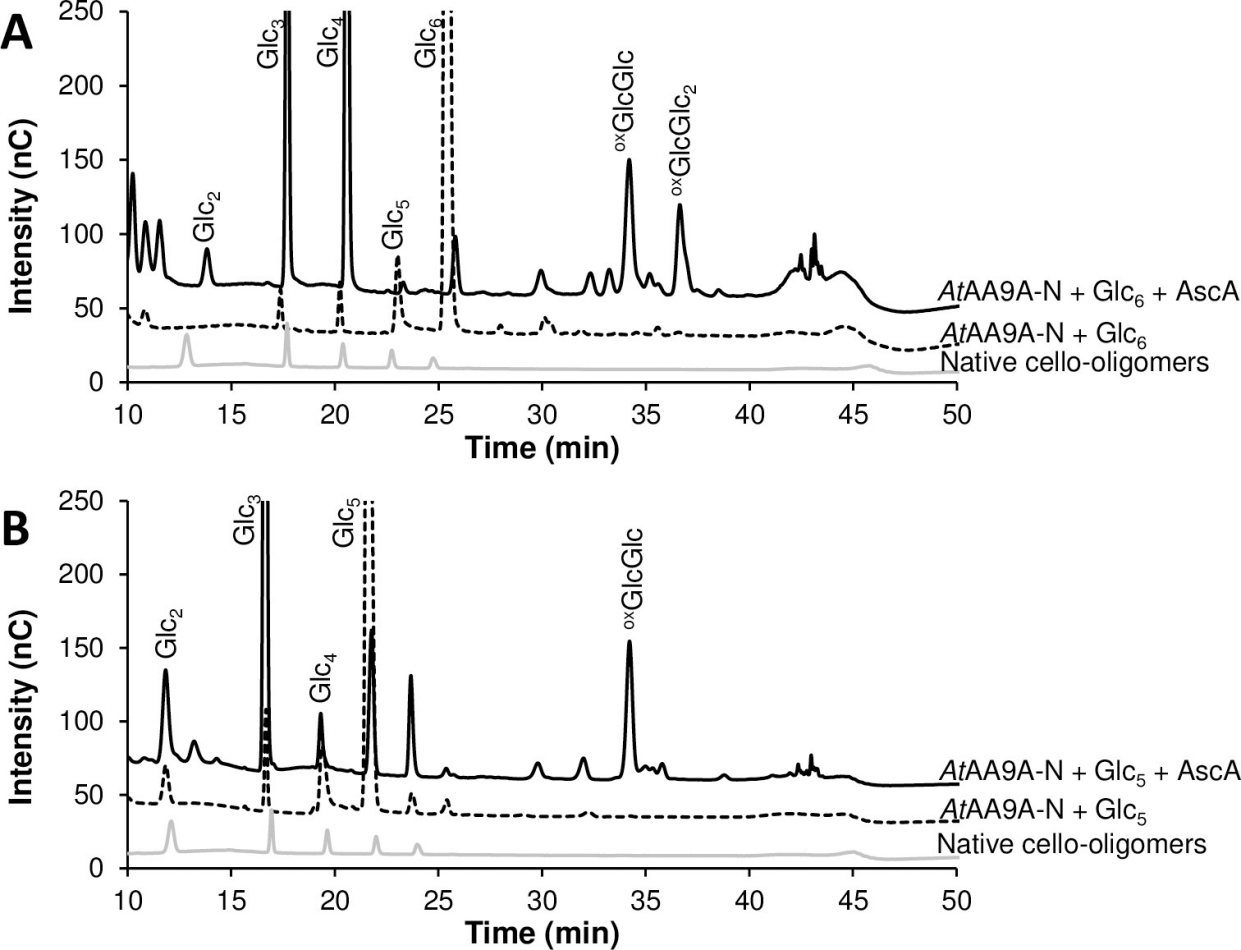
Oxidative cleavage of cellohexaose (Glc_6_) (A) and cellopentaose (Glc_5_) (B) by *At*AA9A-N. The graphs show HPAEC-PAD profiles of reaction mixtures containing 1 μM LPMO and 1% (w/v) oligomeric substrate in 50 mM of BisTris-HCl pH 6.0 with (solid black line) or without (dashed black line) ascorbic acid after incubation at 37°C for 18 h. The grey line shows native cello-oligosaccharide standards with DP 2–6.

Incubation of *At*AA9A-N and *At*AA9B-N with cellohexaose (Glc_6_) and cellopentaose (Glc_5_) showed an absence of activity for *At*AA9B-N (not shown), whereas *At*AA9A-N displayed activity on both cello-oligosaccharides (Fig 2). *At*AA9A-N generated mainly native Glc_4_ and Glc4gemGlc (C4-oxidized with degree of polymerization, DP, 2) and, to a smaller extent, Glc_3_ and Glc4gemGlc_2_ (C4-oxidized DP 3) from Glc_6_, and native Glc_3_ and Glc4gemGlc (C4-oxidized DP 2) from Glc_5_.

**Fig 3:**
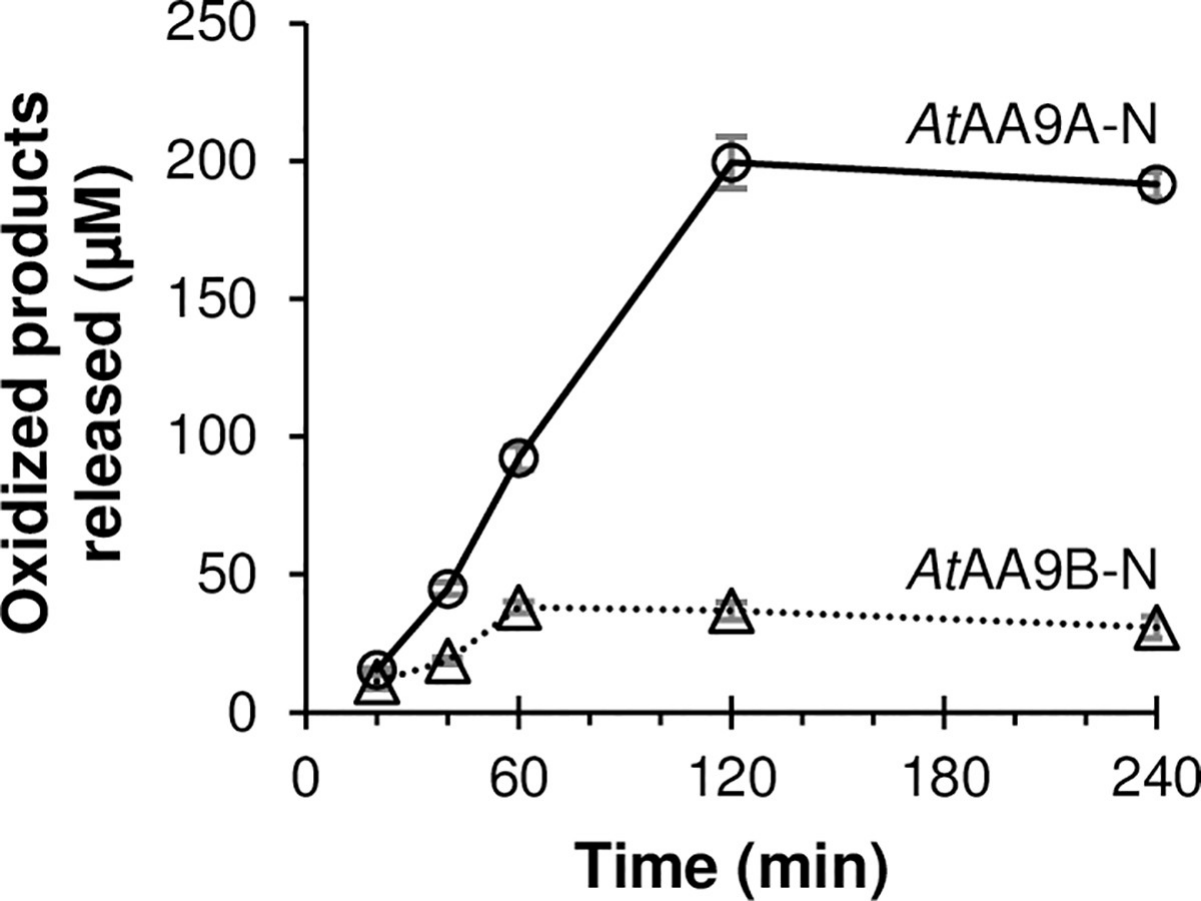
LPMO activity on cellulose over time. The graph shows the accumulation of soluble oxidized oligosaccharides generated from 1% (w/v) PASC by 1μM *At*AA9A-N (solid line with circles) or *At*AA9B-N (dotted line with triangles) in 50 mM of BisTris-HCl pH 6.0 in the presence of 1 mM ascorbic acid, over time. The reactions were incubated at 37°C and samples were collected after 20, 40, 60, 120, and 240 min of incubation.

Product formation by *At*AA9A-N and *At*AA9B-N over time was also assessed using PASC as a substrate. To facilitate quantification of product formation, the soluble products generated by the LPMOs were treated with a cellobiohydrolase, which converts both C1- and C4-oxidized cello-oligosaccharides to the corresponding oxidized dimers (for details, see the Materials and methods). As expected based on previous LPMO studies using the same reaction conditions (e.g. [61, 62]), both enzymes showed a linear phase of product formation followed by termination of the reaction. *At*AA9A-N was faster than *At*AA9B-N and reached a higher yield (*At*AA9A-N, 199±9 μM in 120 min; *At*AA9B-N, 38±2 μM in 60 min). In addition, product formation by *At*AA9B-N leveled off sooner, after 60 min of incubation, while *At*AA9A-N continued to release oxidized oligosaccharides for up to 120 min (Fig 3). The initial rates that can be estimated from the linear parts of the progress curves in Fig 3, 3.3 min^-1^, and 0.6 min^-1^ for *At*AA9A-N and *At*AA9B-N, respectively, are in the same range as the rates of other LPMOs working under the same conditions [63].

### Hemicellulolytic activity of *At*AA9A-N and *At*AA9B-N

**Fig 4:**
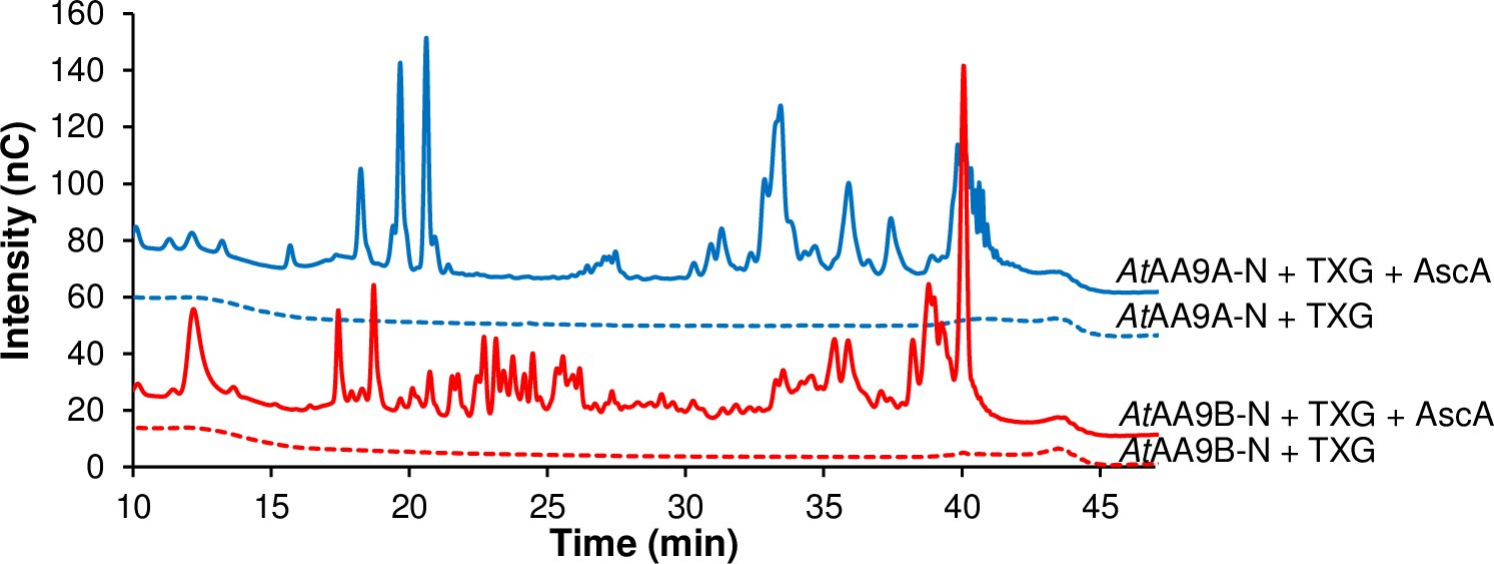
Oxidative cleavage of tamarind xyloglucan (TXG) by *At*AA9A-N and *At*AA9B-N. The graph shows HPAEC-PAD profiles of reaction mixtures containing 1 μM *At*AA9A-N (blue lines) or 1 μM *At*AA9B-N (red lines) and 1% (w/v) TXG in 50 mM of BisTris-HCl pH 6.0, with (solid lines) or without (dashed lines) 1 mM ascorbic acid (AscA) after incubation at 37°C for 18 h.

**Fig 5:**
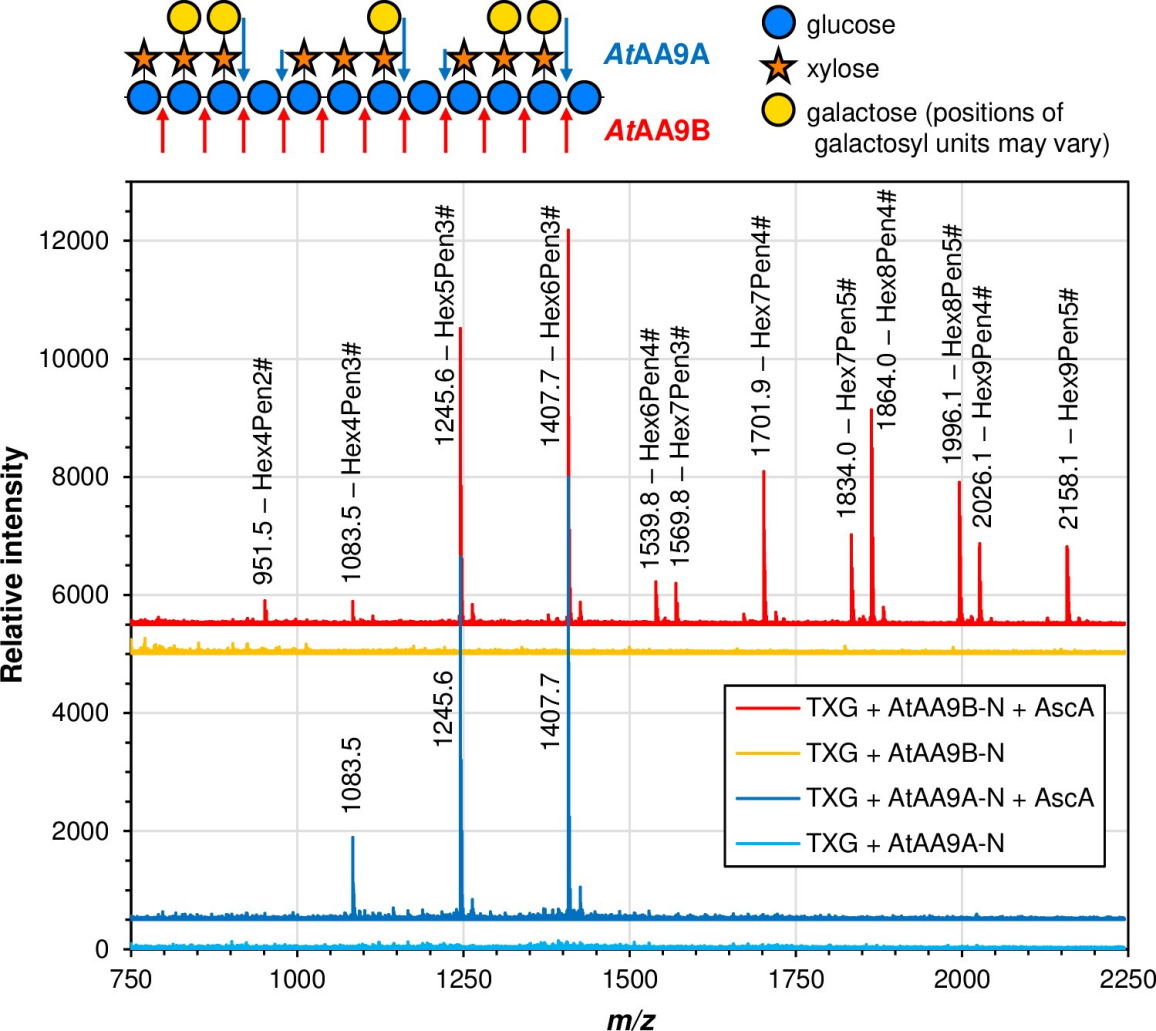
Products generated from xyloglucan. The picture shows MALDI-TOF MS spectra of xyloglucan-oligosaccharides released by *At*AA9A-N (dark blue and light blue lines) and *At*AA9B-N (red and orange lines) from tamarind xyloglucan (TXG). The indicated *m/z* values refer to sodium adducts of singly oxidized species (# indicates oxidation). The reactions contained 1% (w/v) TXG and 1 μM LPMO in 50 mM of BisTris-HCl pH 6.0, with (dark blue and red lines) or without (light blue and orange lines) 1 mM ascorbic acid (AscA). Reactions were incubated at 37°C for 18 h. The picture to the upper left shows xyloglucan and how it may be cleaved by *At*AA9A and *At*AA9B.

*At*AA9A-N and *At*AA9B-N were both able to cleave tamarind xyloglucan but yielded different product mixtures (Figs 4 and 5). While the peaks in the chromatographic profiles could not be annotated due to unavailability of xyloglucan oligosaccharide standards, it is clear that these profiles are very different (Fig 4). The nature of this difference was revealed by MALDI-TOF MS analysis of the reaction products. The xyloglucan backbone contains an unsubstituted glucose (G) every four sugars, whereas the other glucoses are substituted with a pentose, xylose (X), which again may be substituted with another hexose, galactose (L) [17]. *At*AA9A-N produced a clustered product profile typical for enzymes that can cleave xyloglucan only next to the unsubstituted glucose units, yielding for example a cluster of (oxidized) Hex_4_Pen_3_ (e.g. GXXX), Hex_5_Pen_3_ (e.g. GXXL) and Hex_6_Pen_3_ (e.g. GXLL) (Fig 5; [9]). From the current data, it is not possible to say whether, for example, the Hex_4_Pen_3_ product is ^ox^GXXX or ^ox^XXXG but previous detailed studies of *Nc*AA9C, with a substrate-binding surface similar to that of *At*AA9A-N (see below), have shown that this enzyme predominantly cleaves on the non-reducing side of a non-substituted glucose [9] and would thus, in this example, produce ^ox^GXXX. On the other hand, *At*AA9B-N produced a myriad of products indicating that xyloglucan was also cleaved in between substituted glucose units (Fig 5).

For both LPMOs, detected products were almost exclusively oxidized, as illustrated, e.g., by the product cluster for *At*AA9A-N in Fig 5 showing oxidized GXXX (*m/z* 1083.5), GXXL (*m/z* 1245.6), and GXLL (*m/z* 1407.7). The relatively low signals for hydrated oxidized products (e.g. the signal at 1425.7 for GXLL) and the absence of signals representing the sodium salts of aldonic acids both suggest that oxidation of XG happened at C4 only for both LPMOs, although this cannot be concluded with certainty. Apart from activity on xyloglucan, we could not detect activity on the other hemicellulosic substrates tested (lichenan, ivory nut mannan, and birchwood xylan).

## Discussion

Transcriptome analysis of *A*. *tamarii* growing on sugarcane bagasse as a carbon source revealed expression of seven AA9 LPMOs [39]. Of these, five were upregulated during the 48h growth period on this plant biomass, namely *At*AA9A, *At*AA9B, *At*AA9D, *At*AA9E and *At*AA9G [39] (see also S2 Table in S1 Appendix). The differences in domain organization and variations in amino acid sequence indicate distinct roles of these LPMOs in biomass degradation. Here, we report characteristics of the catalytic domains of two of these LPMOs, *At*AA9A, and *At*AA9B.

*At*AA9A and *At*AA9B are both multi-modular enzymes. *At*AA9A has a CBM1, which is commonly found attached to fungal AA9-type LPMOs. *At*AA9B contains a short C-terminal domain of unknown function that seems specific to LPMOs of *Aspergillus* and *Penicillium* species. It is worth noting that the alignment of these small domains of approximately 40 residues (S1A Fig in S1 Appendix) shows a fully conserved Tyr/Trp, Trp, and His residue, i.e. residues that are often seen to contribute to the binding of carbohydrates. The differences in the C-terminal domains of the two proteins suggest that the two enzymes may target different parts of the plant cell wall.

**Fig 6:**
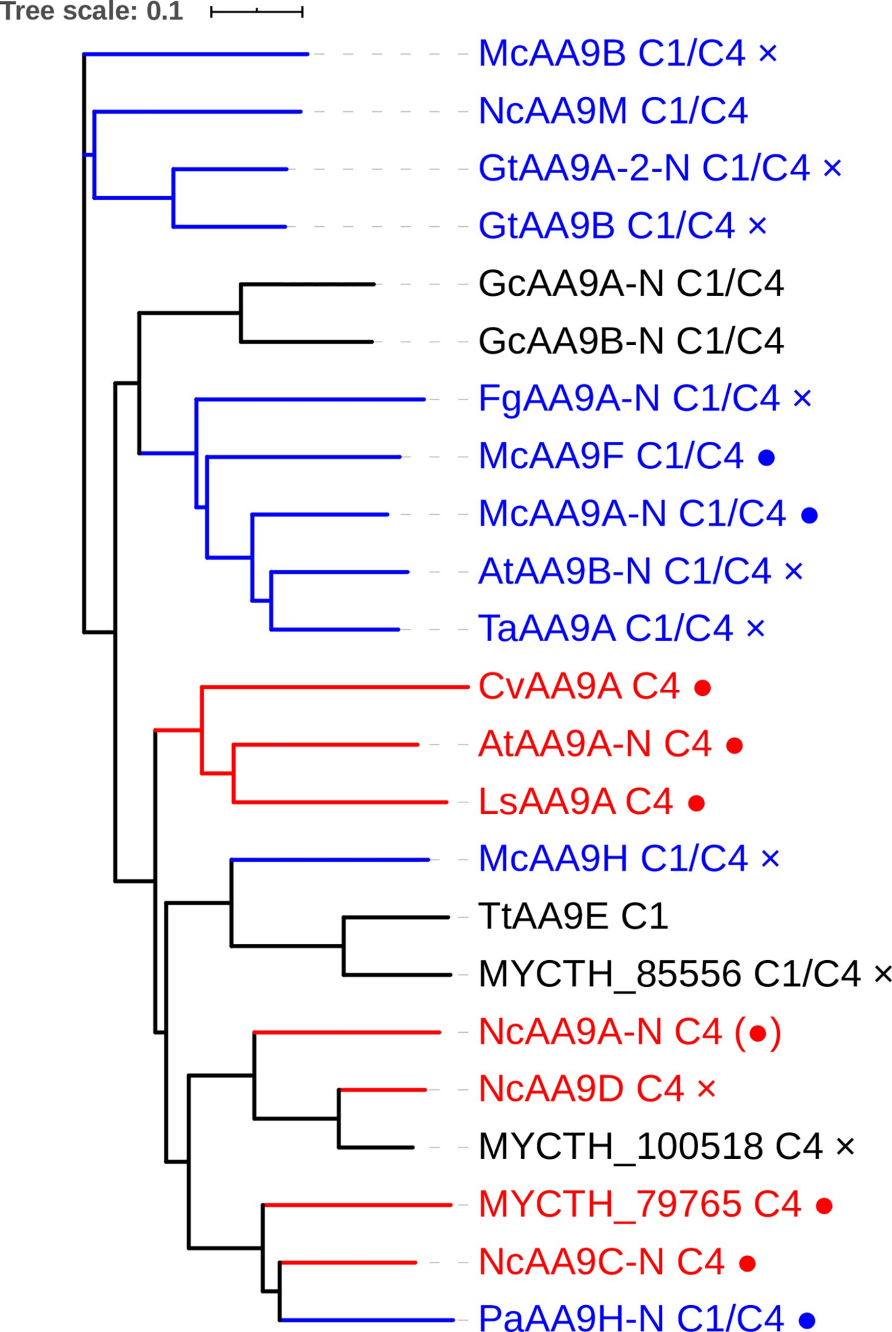
Phylogenetic tree for AA9 LPMOs for which xyloglucan activity has been unequivocally demonstrated. Red labels indicate LPMOs with a substitution-intolerant cleavage pattern, whereas blue labels indicate a less restricted cleavage pattern; black labels indicate that the cleavage pattern is unknown. Regioselectivity on cellulose (C1, C4, or C1/C4) and activity on soluble cello-oligosaccharides (full circle, active; cross, inactive; no sign, unknown) are indicated after the enzyme name. For more information on phylogeny, including less well characterized AA9 LPMOs, see S2 Fig in S1 Appendix.

Here, we have employed cleavage of PASC, soluble cello-oligosaccharides, and xyloglucan as a proxy for identifying functional differences. While *At*AA9A-N cleaved cellulose with C4-oxidation and was active on cello-oligosaccharides (Glc_6_ and Glc_5_), *At*AA9B-N cleaved cellulose with C1/C4-oxidation and was inactive on cello-oligosaccharides. Notably, *At*AA9A-N produced only shorter C4-oxidized cello-oligosaccharides from PASC (Fig 1), which underpins its activity on cello-oligosaccharides, as observed before for *Nc*AA9C [13, 50]. Initially it was postulated that LPMO activity on short cello-oligosaccharides may indicate activity on hemicelluloses that contain glucose in the polysaccharide backbone [50], which was later confirmed in multiple studies (e.g. [9, 10, 64, 65]). On the other hand, several LPMOs have been described that are inactive on soluble cello-oligosaccharides, but that are able to cleave polymeric hemicelluloses [15, 16, 57, 61, 62, 66] (Fig 6 and S4 Table in S1 Appendix). Correspondingly, despite the difference in activity towards cello-oligosaccharides, *At*AA9A-N and *At*AA9B-N both cleaved tamarind xyloglucan. The two *At*AA9s characterized in this study show distinct cleavage patterns: *At*AA9A-N exhibited substitution-intolerant cleavage, whereas *At*AA9B-N exhibited substitution-tolerant cleavage. In general, the properties of *At*AA9A-N resemble those of experimentally and structurally characterized *Nc*AA9C [9, 67], *Ls*AA9A [56] and *Cv*AA9A [64]. The properties of the catalytic domain of *At*AA9B resemble those of experimentally and structurally characterized *Ta*AA9A [2, 57] and *Nc*AA9M [68, 69].

LPMOs are prone to oxidative inactivation [70] and tend to be unstable under commonly used reaction conditions such as those employed here [71]. While in-depth assessment of LPMO stability is beyond the scope of this study, the progress curves with PASC revealed another difference between the two LPMOs: *At*AA9A-N was more stable and gave higher product yields than *At*AA9B-N. In both cases, the final yields (ca. 200 and 40 μM soluble oxidized products, respectively) stayed well below the theoretical maximum, which is defined by the presence of 1 mM ascorbic acid and the degree by which oxidized products become soluble [72]. It has recently been shown that efficient binding to substrate increases the redox stability of LPMOs and that removal of the CBM may lead to increased LPMO inactivation [72–75]. Thus, it is possible that the catalytic domains studied here are less stable than the full-length enzymes. Nevertheless, the difference in the progress curves of Fig 3 add to the notion that the catalytic domains of these two LPMOs have different properties.

Accumulating data for multiple LPMOs active on cello-oligosaccharides and xyloglucan, summarized in Fig 6 and S4 Table in S1 Appendix, now allow for meaningful speculation about the possible structural causes of the varying substrate specificities. In order to understand structural differences behind the distinct xyloglucan cleavage patterns and the ability to cleave soluble cello-oligosaccharides, we first aligned the sequences of xyloglucan-active AA9s with known cleavage types (S5 Fig in S1 Appendix), paying particular attention to the L2, L3, and LC loops that all contribute to shaping the substrate-binding surface [67, 69, 76] for which NMR and crystallographic studies have shown that they contain residues that are involved in binding of substrate [56, 64, 77]. Notably, existing data for LPMO–substrate interactions show that residues putatively involved in substrate binding not only occur in these three loops but also in a region between the LS and LC loops (residues from His147 to Tyr 166 for *Ls*AA9A in S5 Fig in S1 Appendix). This was named “Seg4” in a recent study by Laurent et al. [78], based on the observation that this region is a meaningful discriminator for the phylogenetic grouping of AA9 LPMOs.

**Fig 7:**
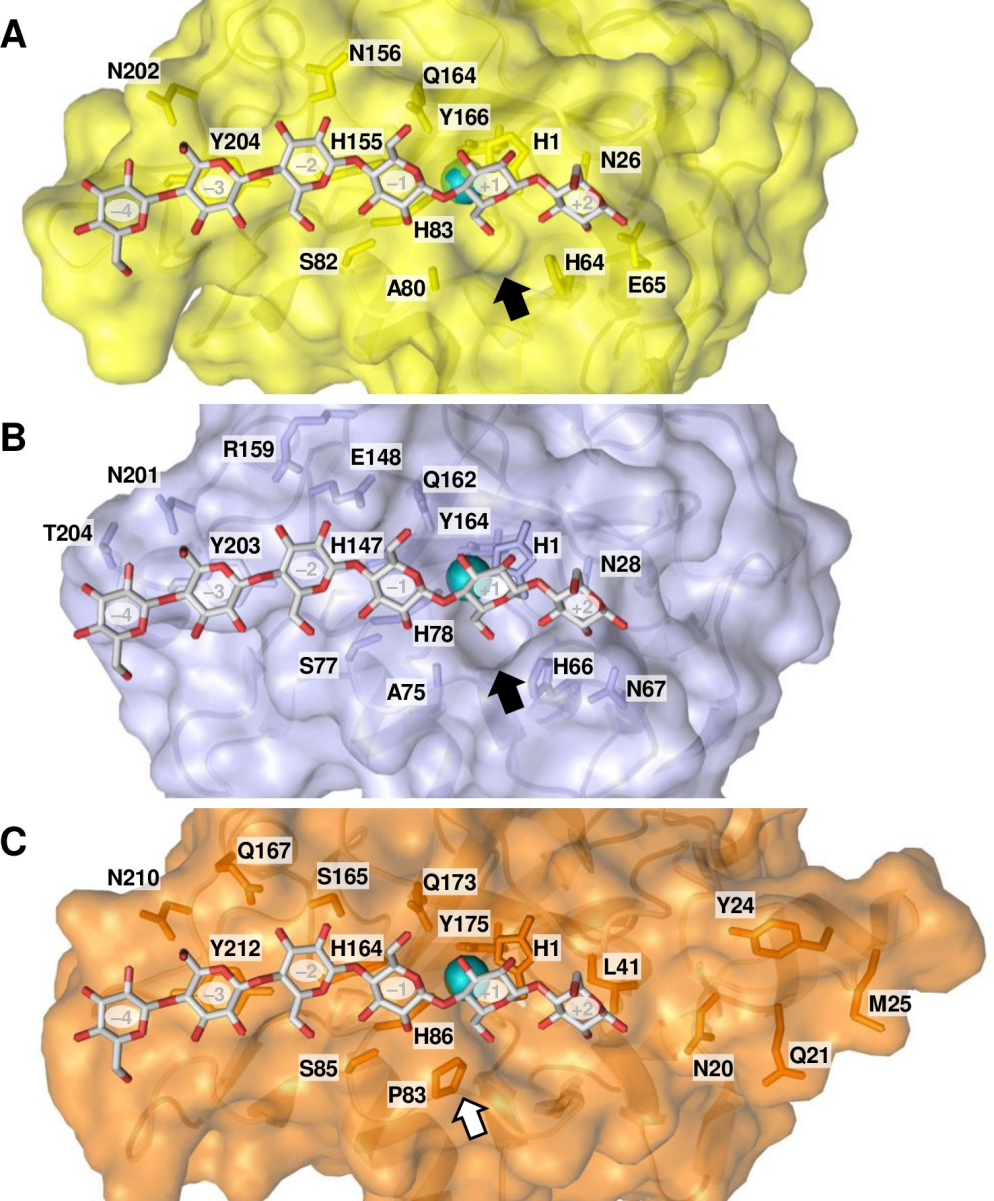
The substrate-binding surface of (A) *Nc*AA9C, (B) *Ls*AA9A and (C) *Ta*AA9A. Panels **A** and **B** show a cavity (black arrow) formed by the L3 loop of **(A)** *Nc*AA9C (PDB: 4D7U) and **(B)** *Ls*AA9A (PDB: 5ACI), both cleaving xyloglucan adjacent to an unsubstituted unit; panel **C** shows the lack of cavity and a conserved surface-exposed proline (white arrow) in *Ta*AA9A (PDB: 2YET), being able to cleave xyloglucan between two substituted units. The cellohexaose was superposed from the *Ls*AA9A–cellohexaose (PDB: 5ACI) structure. The same cavity is present in all other substitution-intolerant AA9 LPMOs with a known crystal structure, *Cv*AA9A (PDB: 5NLT), *Nc*AA9A (PDB: 5FOH) and *Nc*AA9D (PDB: 4EIR). As to other substitution-tolerant AA9 LPMOs, to date structural data is available only for *Nc*AA9M (PDB: 4EIS), which similarly shows the lack of cavity and a conserved surface-exposed proline. Note that panel C shows side chains of residues in the extended L2 region of substitution-tolerant *Ta*AA9A, including aromatic Tyr24. More details of the structures are provided in S6 Fig in S1 Appendix, which shows a structural superposition.

Multiple sequence alignment (S5 Fig in S1 Appendix) of xyloglucan-active AA9s showed that the substitution-tolerant XG-active AA9s have shorter L3 loop regions compared to their more restricted counterparts, with the structural effects of this difference illustrated in Fig 7 as well as in S6 and S7 Figs in S1 Appendix. Crystallographic and NMR studies have shown that the L3 loops of (substitution-intolerant) *Nc*AA9C and *Ls*AA9A carry multiple residues that interact with the substrate, namely His64 and Ala80 in *Nc*AA9C [77] and His66, Asn67, Ala75 and Ser77 in *Ls*AA9A [56]. The crystal structure of the *Ls*AA9A–cellohexaose complex (PDB: 5ACI) reported by Frandsen et al. [56] revealed that the C6 hydroxyl of the glucose at subsite +1 is accommodated in a small pocket that is largely shaped by residues in the L3 loop, namely His1, His66, and Ala75, corresponding to His1, His64, and Ala80 in *Nc*AA9C (Fig 7). This pocket is too small to accommodate a glucose with a xylosyl substitution at C6, as it would occur in xyloglucan [17]. In agreement with this, Agger et al. [9] concluded that *Nc*AA9C converts the xyloglucan-oligosaccharide XG14 primarily to XXX and ^ox^GXXXG, which implies that cleavage occurs when an unsubstituted glucose is bound to the +1 subsite. Recently, Sun et al. confirmed that *Nc*AA9C cleaves polymeric XG predominantly by non-reducing side of a non-substituted glucose [69]. Notably, this pocket is lacking in substitution-tolerant XG-active AA9 LPMOs that have a shorter L3 loop and in which, with one exception (*Mc*AA9H), the Ala75/80 is replaced by a proline (Fig 7 and S5 Fig in S1 Appendix). This proline (or Tyr in *Mc*AA9H), which is part of a more open substrate-binding surface, may interact with a xylosyl moiety at subsite +1. Expectedly, the structural models of *At*AA9A and *At*AA9B (S7 Fig in S1 Appendix) correspond to their template structures *Ls*AA9A (PDB ID, 5N05) and *Ta*AA9A (PDB ID, 3ZUD), respectively, predicting a similar pocket formed by the corresponding residues in the L3 loop of *At*AA9A and a surface-exposed proline in *At*AA9B.

Next to having a sterically less restrained +1 subsite, the substitution-tolerant xyloglucan-active AA9s contain insertions in their L2 loop that are absent in substitution-intolerant xyloglucan-active AA9s. Sun et al. recently suggested that this extension in the L2 loop (referred to as Seg1) combined with a shorter L3 loop (referred to as Seg2) may correlate with substitution-tolerant cleavage of XG [69]. While there is little experimental data in support of involvement of the L2 region in substrate binding, such involvement seems obvious from looking at available LPMO structures (Fig 7C and S8 Fig in S1 Appendix). The region with insertions (residues 16–40 for *Ta*AA9A) carries one or two aromatic (Tyr or Phe) residues (S5 Fig in S1 Appendix). The structures of *Ta*AA9A (Fig 7C) and *Nc*AA9M, the only substitution-tolerant xyloglucan-active AA9s with a resolved crystal structure [2, 68], show that these aromatic residues may be surface exposed and thus contribute to binding of polymeric substrates. An extended substrate-binding surface could facilitate binding of a multitude of substrates, since the mere size of the interacting surface may compensate for sub-optimal interactions in one or a few subsites. Of note, while the correlations above seem quite general, and are supported by a recent comparative study by Sun et al. [69], *Pc*AA9H is a notable exception, since its activity on XG is substitution-tolerant, while its sequence and predicted structure resemble that of substitution-intolerant XG-degrading LPMOs (Fig 6 and S5 and S8 Figs in S1 Appendix).

It is worthwhile noting that the substitution-intolerant xyloglucan-cleaving LPMOs cleave at C4, whereas the substitution-tolerant enzymes tend to show C1 and C4 oxidation (Fig 6 and S4 Table in S1 Appendix). Regioselectivity on xyloglucan has been confirmed unambiguously only for a handful of LPMOs, and the available data suggest that regioselectivity on xyloglucan corresponds to regioselectivity on cellulose [9, 15, 69, 79]. This adds to the notion that substitution-intolerant LPMOs have more restrained subsites that lead to tight and precise binding close to the catalytic copper, whereas substitution-tolerant LPMOs bind their substrate in a manner that is not disturbed by the substitutions present, leading to mixed oxidation patterns. In this respect, one might expect that the substitution-intolerant enzymes, with their smaller but potentially tighter binding substrate-binding surfaces, would be the only ones acting on soluble cello-oligosaccharides. However, while, indeed, compared to the substitution-tolerant LPMOs, a larger fraction of substitution-intolerant LPMOs cleaves soluble oligomers (Fig 6 and S4 Table in S1 Appendix), the correlation between the type of xyloglucan cleavage and the ability to cleave oligomers is far from absolute and may even not exist. As yet, it seems not possible to make meaningful predictions regarding the structural features that determine activity on soluble substrates. Such predictions await structural information for more LPMOs and more LPMO–substrate complexes.

In summary, we show that two of the AA9 LPMOs from *A*. *tamarii* have distinct substrate and product profiles, which corroborates that filamentous fungi have evolved LPMOs with diversified substrate specificities and oxidative regioselectivities and that these LPMOs likely complement each other in natural biomass degradation. Fungi are singular microorganisms that are adapted to multiple ecological niches and different conditions, thus displaying a potential for innumerable applications. Gaining a better knowledge of their enzymatic repertoire is crucial for exploiting and (re)designing their biotechnological applications.

## Supporting information

S1 Appendix (PDF)
