# Supplementary material for: Characterization of two family AA9 LPMOs from Aspergillus tamarii with distinct activities on xyloglucan reveals structural differences linked to cleavage specificity
Source: PLoS One. 2020 Jul 8;15(7):e0235642. doi: 10.1371/journal.pone.0235642 (PMC7343150; doi:10.1371/journal.pone.0235642)
Supplement: S1 Appendix — (PDF) [file pone.0235642.s001.pdf]

S1 Appendix

Supporting information

for

Characterization of two family AA9 LPMOs from *Aspergillus tamarii* that are active on xyloglucan

Antonielle V. Monclaro[1,2], Dejan M. Petrović[2], Gabriel S. C. Alves[3], Marcos M. C. Costa[4], Glaucia E. O. Midorikawa[3], Robert N. G. Miller[3], Edivaldo X. F. Filho[1], Vincent G. H. Eijsink[2], Anikó Várnai[2,*]

List of content

Supplementary tables

S1 Table. Sequences of putative AA9 LPMOs from *Aspergillus tamarii* BLU37.

S2 Table. Comparison of AA9 LPMOs found in the *A. tamarii* BLU37 transcriptome with putative AA9 LPMOs encoded in the genomes of *A. oryzae* RIB40 and *A. tamarii* CBS 117626, and identification of experimentally characterized LPMOs with the highest sequence identity.

S3 Table. Domain structure and predicted properties of the *A. tamarii* AA9 LPMOs.

S4 Table. Regioselectivity and substrate specificity of *At*AA9A-N, *At*AA9B-N, and experimentally characterized AA9 LPMOs that are active on xyloglucan.

Supplementary figures

S1 Fig. Consensus and frequency of amino acids in the putative C-terminal domains of (A) *At*AA9B and (B) *At*AA9C.

S2 Fig. Phylogenetic tree of the AA9 domains of *A. tamarii* AA9s with characterized AA9 LPMOs.

S3 Fig. SDS-PAGE of the purified recombinant *At*AA9A-N and *At*AA9B-N.

S4 Fig. MALDI-ToF spectra showing products generated upon incubation of *At*AA9A-N and *At*AA9B-N with PASC.

S5 Fig. Multiple sequence alignment of the catalytic domains of AA9 LPMOs for which activity on xyloglucan has been demonstrated.

S6 Fig. Structural superposition of (substitution-intolerant) *Nc*AA9C-N, *Ls*AA9A, and *Cv*AA9A and (substitution-tolerant) *Ta*AA9A, showing surface-exposed side chains that (potentially) take part in protein–substrate interactions.

S7 Fig. The predicted substrate-binding surface of (A) *At*AA9A-N and (B) *At*AA9B-N.

S8 Fig. Sideview of xyloglucan-active LPMOs which have (A) substitution-intolerant, (B) substitution-tolerant, or (C) unknown cleavage pattern.

Supplementary tables

S1 Table. Sequences of putative AA9 LPMOs from *Aspergillus tamarii* BLU37. The sequences were derived from earlier RNA-sequencing of the transcriptome of *Aspergillus tamarii* BLU37 [1]. Regions annotated as AA9 domains, using the dbCAN2 metaserver (http://bcb.unl.edu/dbCAN2/) and by structural alignment with so far characterized AA9 LPMOs using T-Coffee's Expresso tool (http://tcoffee.crg.cat/apps/tcoffee/do:expresso), are marked in blue; regions annotated as CBM1 domains are marked in green; putative C-terminal domains in *At*AA9B and *At*AA9C are marked in red and orange, respectively (for details, see S1 Fig). N-terminal signal peptides and non-annotated regions, including regions of low sequence complexity that may be flexible linkers, appear in black. Amino acids that represent deviations from the predicted AA9 sequences in *A. tamarii* CBS 117626 [2] are highlighted in grey. (Note that the C-terminal sequences of *At*AA9D and *At*AA9F are truncated compared to the corresponding proteins in *A. tamarii* CBS 117626.)

```
>AtAA9A
MKSSTFGMLALAAAAKLVSAHTTVHAVWINDVDQGEGNSQSGYIRSPPSNSPITDVTSKDMTCNVNNKAT
AKTLEVKAGDKITFEWHHDSRSESDDIIASSHNGPILVYMAPTEKGTAGNGWVKIAEDGYTDGTWAVETL
IKNRGKHSVTVPDVAAGEYLFRPEIIALHEGNREGGAQFYMECVQVKVTSSGSKTLPEGVSIPGAYTATD
KGILFNIYDSFDSYPIPGPAVWDGASGSSSSSSSASASAPAPTSAAPAPSSFTTIAKQPATSSSTEAPS
TENTPSETTSTTSAIVSTTAVASTTAPATPSTTSAIASSAAPTNSVPQPSSNAGGAVKEWYQCGGLNYSG
STQCEEGLTCKKWNPYYHQCVSA
```

```
>AtAA9B
MSIAKIAGVVLGSAALVAGHGYVSGAVVDGQYYSGYDMSYHYMSDPPKVIGWSTDATDLGFVDGSSYADA
DIICHKNAKNGAISAEIAAGKQVELQWTDWPESHKGPVITYLANCNGDCATVDKTQLEFFKIDEKGLISG
SDNTWASDNLISSNNSWTVTIPSSIAAGNYVMRHEIIALHSAGNKDGAQNYPQCLNFKVTGGGSDKPEGT
LGTALYKDTDPGILVNIYQTLSSYTIPGPALYSGSSSGSSSGSSGSSSAAPSATASASASATAAPVQTST
ATAYQTSTAVASVTVTGSAPAQTHVQATSSSAAASTPTASSGASSGSGSSSSSSSSDLTDYFNSLSADEL
LNVIKQTLSWLVTDKIHARDISA
```

```
>AtAA9C
MFRSALFLLLAPLALSHTTFTTLYVDEVNQGDGTCVRMNRDANTVTYPIEPLSSKDIACGKDGEKAVSRV
CPAKANSLLTFEFRAWADGAQPGSIDISHKGPCAVYMKKVDDATADNNAAGDGWFKIWHTGYDESTEKWC
TEKLIDNNGFLSVRVPSDIEQGYYLVRTELLALHAASDAPPDPQFYVNCAQIFVQGGGSAKPETVSIGEG
YYSLDSPGVKYNIYEKPLQLPYPIPGPTVYESKGVEERSVCPAQKRTATAQNKGLKPAGCILQRDNWCGF
EVPDYSDENGCWAVCSSFPYQDFMVNTNSLLVIQEVLGSE
```

```
>AtAA9D
MKLSLLAIAAIAPFVSAHYFFDTLIIDGQESSPNQYVRSNTRAAKYNPTKWVNTRDNMTPDMPDFRCNKG
AFTFAGQTGTAEVKAGSKLALKLGVGATMKHPGPALVYMSKAPSTAKTYQGDGDWFKIYEEGVCDKNKDL
KSDAWCSWDKDRVEFTIPADLPDGEYLIRPEHIGVRVHGAHAGEAEFYYR
```

```
>AtAA9E
MAMSKIMSLTGLLASASLVAGHGYVSGVAAAYGGYLVDKYAYSDNPPETIGWSTTATDLGFVDGTGYQSP
DIICHKDGKPGALSAEVAAGGEIELQWTEWPESHHGPVLNYLAPCGGDCSAVDKTSLEFFKIEAKGLIDG
SSPPGHWATDDLISNNNSWTVTIPASVQEGNYVLRHEIIGLHSAGQKDGAQNYPQCINIKVTGGGAATPA
GTAGEALYKDTDPGILFDIYSDLSGGYPIPGPEVFSA
```

>AtAA9F
MRHVQSASLLTALLSATKVAAHGHVSNIVINGVYYEGFDINSFPYMGENAPTVAAWTTPNTGNGPLAPDD
YSSPDIICHQNATAGKGYVEVNAGDRISLQWTPWPESHHGPVVDYLARCEPNCASVDKTSLEFFKIDGVG
IVDGSSVPGVWGDDQLIKNNNTWLVEIPKSIAPGYYVLRHELIALHSAGTEGGAQNYPSCFNLKVNGDGT
DKPAGVVGTELYTPTGDGIIFNIYQTVSSYPVPGPTLYTGAATGVTQATSAITSTGTALTVGAAATTPAS
GSGASSSAAPSSSAAATPSSRLSSLCCCVPL

>AtAA9G
MKLNLASLCFLASIAPLVSGHYVFSKLIVDGKTTKDFEYIRENSNGYQPTLASEIVSNDFRCNKGSMESA
AKTKVYTVAPGAEMGFQLAYGASMKHPGPLQIYMSKAPGDVKAYDGSGDWFKVYQEGVCNDISGGLKDTD
WCTWGKDTASFKIPENTPPGQYLVRVEHIGLHRGFSGNSEFYFTCAQIEVTGSGSGVPGPLVKIPGVYKP
EDPNIHFNIYHPVPTSYDLPGPSVWSGGVSDSSSSISAPPVNNAAAASSVTPTTLVTLSKTSSTPAATSS
AAPTSSAPSNGTIKKYYQCGGQGWTGSGSCEAGTSCREWNTWYFQCV

S2 Table. Comparison of AA9 LPMOs found in the A. tamarii BLU37 transcriptome with putative AA9 LPMOs encoded in the genomes of *A. oryzae* RIB40 and *A. tamarii* CBS 117626, and identification of experimentally characterized LPMOs with the highest sequence identity. AA9 LPMOs that have been identified in the transcriptome of *A. tamarii* BLU37 during growth on sugar cane bagasse [1] are compared to AA9 LPMOs that have been identified in the genomes of *A. oryzae* RIB40 [3] and *A. tamarii* CBS 117626 [2]. The closest (partially) characterized LPMOs from *Aspergillus* species are provided in the footnotes.

**Protein from *A. tamarii* BLU37 transcriptome**	**Protein ID of the corresponding protein in the *A. oryzae* RIB40 genome**	**Protein ID in the *A. tamarii* CBS 117626 (Asptam1) genome**	**Closest characterized relatives [a]**	**Upregu-lation after 36 h [h]**	**Upregu-lation after 48 h [h]**
*At*AA9A	1567	254541	*Ls*AA9A [4]; 58% [b]	4.7	5.3
*At*AA9B	2234	140265	*Ta*AA9A [5, 6]; 71% [c]	0.78	2.1
*At*AA9C	4102	258171	– [d]	1.4	1.1
*At*AA9D	4194	312543	– [d,e]	7.0	6.7
*At*AA9E	4749	218852	*Ta*AA9A [5, 6]; 69% [f]	10.5	11.0
*At*AA9F	5772	312044	*Tr*AA9A [7-9]; 58% [g]	1.9	-1.0
*At*AA9G	11276	288991	– [c]	5.5	6.1
– [i]	9997	273838 [i]		– [i]	– [i]
– [i]		303761 [i]		– [i]	– [i]

[a] Enzymes with known crystal structures for the catalytic domain; the sequence identity for the catalytic domain is indicated after the enzyme name.

[b] Of the reported LPMOs, *At*AA9A-N shares 80% sequence identity with An1602, for which C4-oxidizing activity on cellulose has been demonstrated [10].

[c] The closest experimentally characterized relatives of *At*AA9B are Aspte3, Aspfu3, and Chacr2 [11] with 80-84% sequence identity; the regioselectivity of these LPMOs has not yet been identified beyond doubt and data for xyloglucan are lacking.

[d] No sequence identity >50% with an experimentally characterized LPMO.

[e] Of the reported LPMOs, *At*AA9D-N shares 84% sequence identity with An3046-N; the regioselectivity of this enzyme is not known; experiments with non-purified enzymes suggest activity on cellulose and xyloglucan [12].

[f] The closest experimentally characterized relatives of *At*AA9E are Aspfu5 [11] and *Af*AA9B [13, 14] with 72-73% sequence identity; the regioselectivity of these LPMOs has not yet been identified and experiments with xyloglucan were not reported.

[g] The closest experimentally characterized relatives of *At*AA9F are Aspte5, Aspfu2, and Chacr1 [11] with 71-73% sequence identity; the regioselectivity of these LPMOs has not been identified beyond doubt and data for xyloglucan are lacking.

[h] transcript levels during growth on sugar cane bagasse. The numbers show log2 fold change of differentially expressed genes when comparing transcript accumulation using steam-exploded bagasse or glucose as carbon source and are from Midorikawa et al. [1]. Significant upregulation is highlighted by the orange color.

[i] AA9 LPMOs present in the genome of *A. tamarii* CBS 117626 for which no corresponding AA9 have been found in the *A. tamarii* BLU37 transcriptome.

S3 Table. Domain structure and predicted properties of the *A. tamarii* AA9 LPMOs. Full-length and AA9 domains only. Domains, as marked in S1 Table, were annotated by HMMER analysis using the dbCAN2 metaserver (http://bcb.unl.edu/dbCAN2/) and by structural alignment with experimentally characterized AA9 LPMOs using T-Coffee's Expresso tool (http://tcoffee.crg.cat/apps/tcoffee/do:expresso). The other listed characteristics were calculated using Expasy's ProtParam tool (https://web.expasy.org/protparam/).

**Protein**	**Domain structure**	**Full-length enzyme [b]**			**AA9 domain (amino acids)**		
		**Length (amino acids)**	**Molecular mass (kDa)**	**pI**	**Length (amino acids)**	**Molecular mass (kDa)**	**pI**
*At*AA9A	AA9–linker–**CBM1**	353	36.68	5.00	214	23.10	4.97
*At*AA9B	AA9–linker–**unknown1**	354	36.32	4.68	225	24.10	4.66
*At*AA9C	AA9–linker–**unknown2** [c]	325	35.54	4.66	247	26.93	4.66
*At*AA9D	AA9 [a]	173					
*At*AA9E	AA9	226	23.65	4.41	226	23.65	4.41
*At*AA9F	AA9–linker	290	29.96	4.69	229	24.53	4.60
*At*AA9G	AA9–linker–**CBM1**	307	32.75	6.09	217	23.75	5.86

[a] fragment only.
[b] without the signal peptide.
[c] potentially truncated at the C-terminus; see main text for details.

S4 Table. Regioselectivity and substrate specificity of *At*AA9A-N, *At*AA9B-N, and experimentally characterized AA9 LPMOs that are active on xyloglucan. LPMOs for which there is structural data are highlighted in yellow. Regarding the activity on cello-oligosaccharides, the degree of polymerization of the tested oligomers and activity levels against those are listed. Activity on other hemicellulosic substrates, whether it has been tested (reported/not reported) and found (+/–), are also given.

Enzyme	Domain	Regio-selectivity on cellulose	Cello-oligomers[d]	L3 loop	Cleavage type on xyloglucan	Other hemi-celluloses	Reference
*Nc*AA9C	AA9–CBM1	C4	+ / 5-6<br>(+) / 4	+	substitution-intolerant	reported; +	[15]
*Fg*AA9A	AA9–[…][a]	C1/C4	– / 3-6	–	substitution-tolerant	reported; –	[16]
*Gt*AA9A-2	AA9–CBMx[b]	C1/C4	– / 5-6	–	substitution-tolerant	reported; –	[17]
*Ta*AA9A	AA9 only	C1/C4	– / 5	–	substitution-tolerant	reported; –	[6]
MYCTH_79765	AA9 only	C4	+ / 5-6	+	substitution-intolerant	reported; +	[18]
*Gt*AA9B	AA9 only	C1/C4	– / 5-6	–	substitution-tolerant	reported; –	[19]
*Ls*AA9A	AA9 only	C4	+ / 4-6	+	substitution-intolerant	reported; +	[20]
*Cv*AA9A	AA9 only	C4	+ / 4-6	+	substitution-intolerant	reported; +	[20]
*Pa*AA9H	AA9–CBM1	C1/C4	+ / 5	+	substitution-tolerant	reported; +	[21, 22]
MYCTH_85556	AA9 only	C1/C4	– / 1-5	–	?[c]	reported; +	[23]
MYCTH_100518	AA9 only	C4	– / 1-5	+	?[c]	reported; +	[24]
*Nc*AA9A	AA9–CBM1	C4	(+) / 5	+	substitution-intolerant; ((+))[d]	reported; –	[25]
*Nc*AA9D	AA9 only	C4	– / 5	+	substitution-intolerant; (+)[d]	reported; –	[25]
*Mc*AA9A	AA9–[…][a]	C1/C4	+ / 6	–	substitution-tolerant	reported; +	[26]
*Mc*AA9B	AA9 only	C1/C4	– / 6	–	substitution-tolerant	reported; +	[26]
*Mc*AA9F	AA9 only	C1/C4	+ / 6	–	substitution-tolerant	reported; +	[26]
*Mc*AA9H	AA9 only	C1/C4	– / 6	–	substitution-tolerant[e]	reported; +	[26]
*Gc*AA9A	AA9 only	C1/C4	not reported	–	?[c]	not reported	[27]
*Gc*AA9B	AA9–[…][a]	C1/C4	not reported	–	?[c]	not reported	[27]
*An*3046	AA9–[…][a]	?[c]	not reported	–	?[c]	not reported	[12]
*Tt*AA9E	AA9 only	C1	not reported	–	?[c]	not reported	[28]
*Nc*AA9M	AA9 only	C1/C4	not reported	–	substitution-tolerant	not reported	[29]
*At*AA9A-N	AA9–CBM1	C4	+ / 5-6	+	substitution-intolerant	reported;–	this study
*At*AA9B-N	AA9–[…][a]	C1/C4	– / 5-6	–	substitution-tolerant	reported;–	this study

[a] unidentified C-terminal extension

[b] unclassified carbohydrate-binding module (CBM)

[c] unclear; it cannot be determined from the available data

[d] activity levels are indicated as: –, inactive; ((+)), hardly active; (+) little active; +, active

[e] activity on xyloglucan was only found when co-incubating xyloglucan and cellulose

Supplementary figures

```
AtAA9B      314 SSSSDLTDYFNSLSADELLNVIKQTLSWLV-TDKIHARDISA--------- 353
Consensus                ::.*:.... :. :  *. **.  .* ***
```

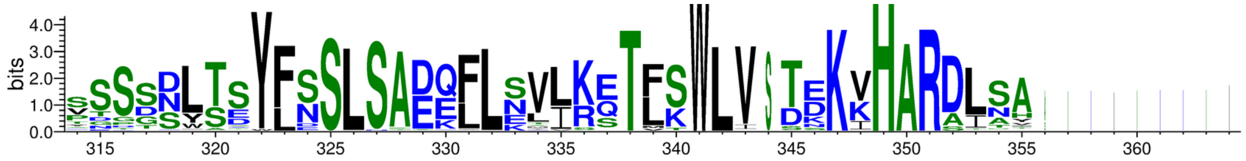

S1A Fig. Consensus and frequency of amino acids in the putative C-terminal domain of *At*AA9B. Consensus was based on multiple sequence alignment of the putative C-terminal domain of *At*AA9B (“unknown 1” in S3 Table) with similar domains in other proteins in the UniProt database; the frequency of amino acids was visualized using WebLogo 3 (http://weblogo.threeplusone.com/create.cgi). Altogether 98 proteins were found (with an E-value <0.0001) when blasting the C-terminus of *At*AA9B (top of the figure) against the UniProt database. All these proteins were identified as AA9 LPMOs using the dbCAN2 metaserver (http://bcb.unl.edu/dbCAN2/). Notably, 96 of the 98 proteins were from *Aspergillus* or *Penicillium* species. The conserved aromatic residues Y322 (Y in 96 and W in 2 sequences), W341, and H348 are highlighted in yellow.

```
AtAA9C-C    248 KGVEERSVCPAQKRTATAQNKGLKPAGCILQRDNWCGFEVPDYSDENGCWAVCSSFPYQD 307
Consensus       *** :***.**:**:.:********************************** ...   *.

AtAA9C-C    308 FMVNTNSLLV--------------IQEVLGSE 325
Consensus        :   .:* .              *::.  *
```

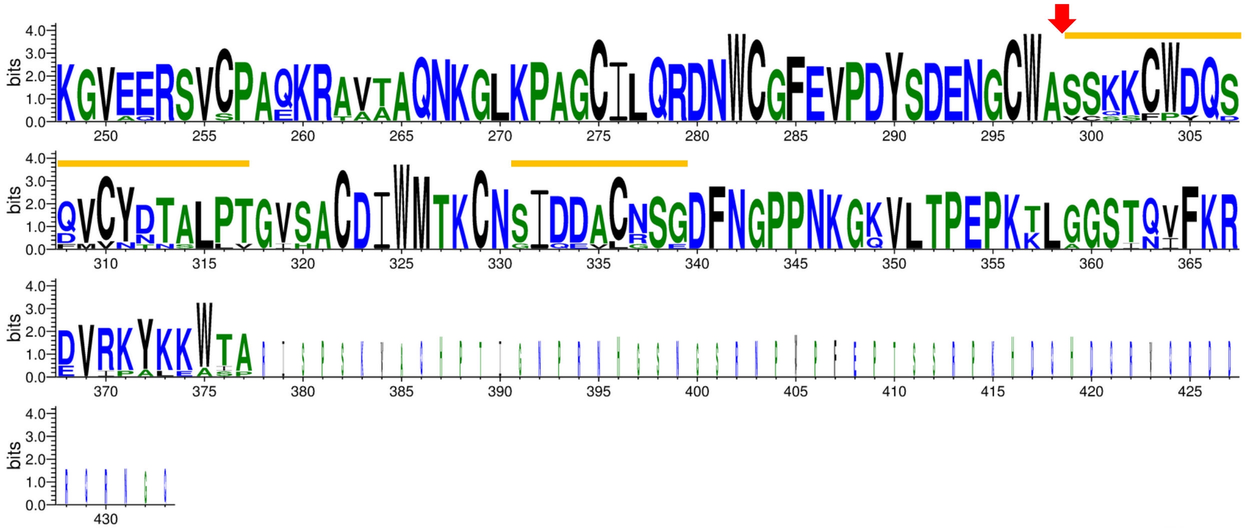

S1B Fig. Consensus and frequency of amino acids in the putative C-terminal domain of *At*AA9C. Consensus was based on multiple sequence alignment of the putative C-terminal domain of *At*AA9C ("unknown 2" in S3 Table) with similar domains in other proteins in the UniProt database; the frequency of amino acids was visualized using WebLogo 3 (http://weblogo.threeplusone.com/create.cgi). Altogether 136 proteins were found (with an E-value <0.0001) when blasting the C-terminus of *At*AA9C (top of the figure) against the UniProt database. All these proteins were identified as AA9 LPMOs using the dbCAN2 metaserver (http://bcb.unl.edu/dbCAN2/), with the exception of one, which is an LPMO fragment (based on multiple sequence alignment). This figure is based on the alignment of the C-termini of *At*AA9C and eight proteins (i.e. the proteins with >90% sequence identity) in the UniProt database. The C-terminal sequence of *At*AA9C seems to be truncated compared to the sequences of these proteins (with >90% sequence identity); the LPMOs shown in the sequence logo are approximately 50 amino acids longer. The putative truncation point in *At*AA9C is indicated by a red arrow and the sequence after the truncated point by orange bars above the sequence and the sequence logo.

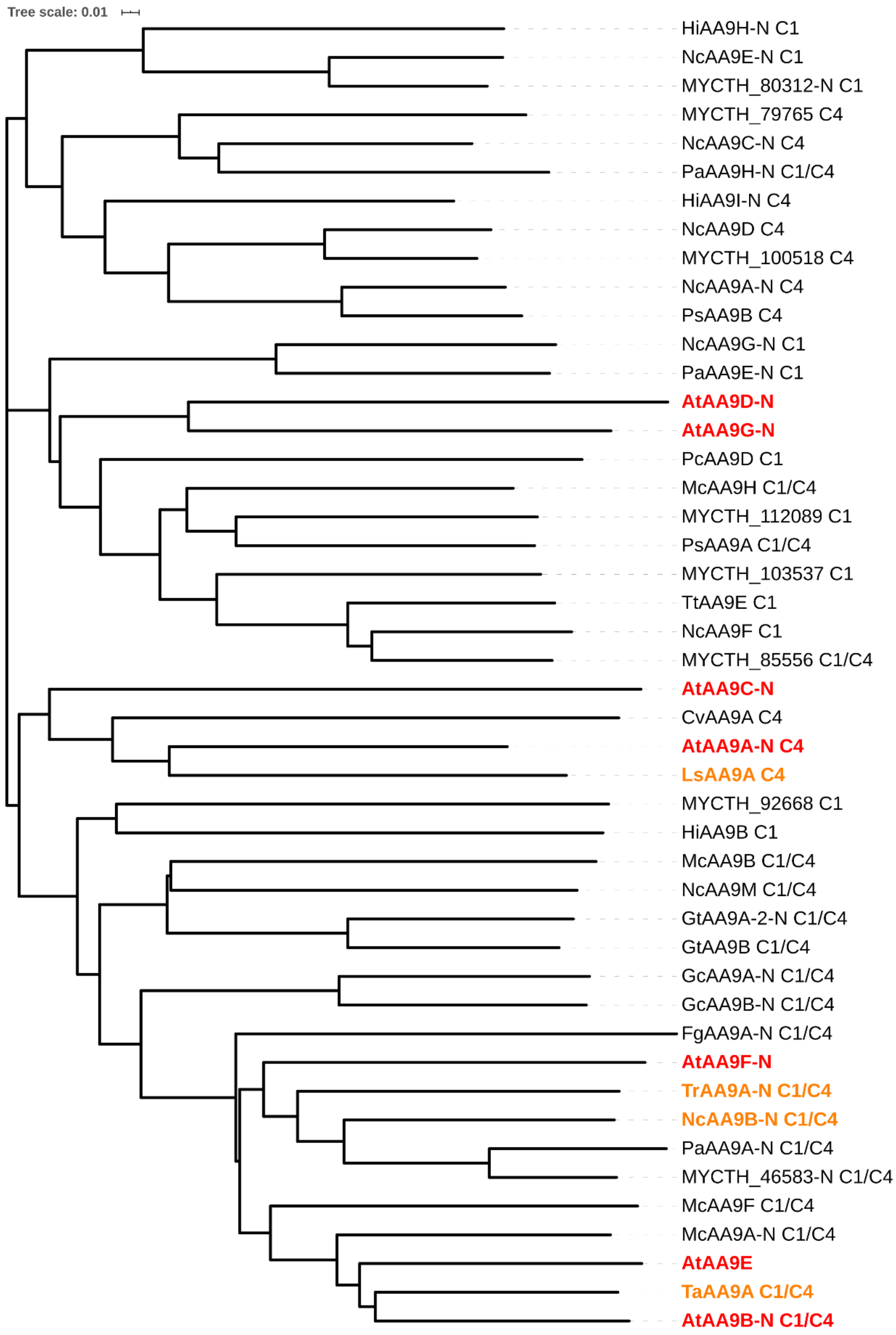

S2 Fig. See legend on the next page.

S2 Fig. Phylogenetic tree based on a multiple sequence alignment of the AA9 domains of *A. tamarii* AA9s with characterized AA9 LPMOs. LPMOs where the reported data were unclear or insufficient to unambiguously identify regioselectivity on cellulose were omitted from the figure. An "-N" after the LPMO name indicates the presence of a C-terminal extension that was omitted from the comparison. *At*AA9s are indicated in red and bold; the closest characterized relatives of the *At*AA9s are indicated in orange and bold. Regioselectivity on cellulose (C1-, C4- and C1/C4-oxidizing) is given after the name of each LPMO.

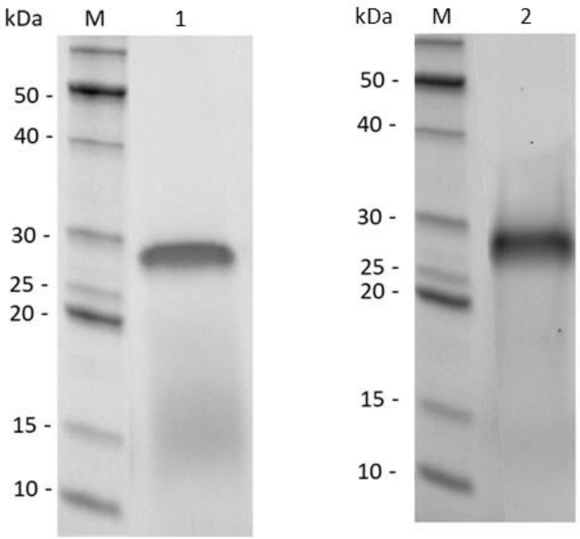

S3 Fig. SDS-PAGE of the purified recombinant *At*AA9A-N and *At*AA9B-N. Lane M, Benchmark Protein Ladder; Lane 1, purified *At*AA9A-N; Lane 2, purified *At*AA9B-N.

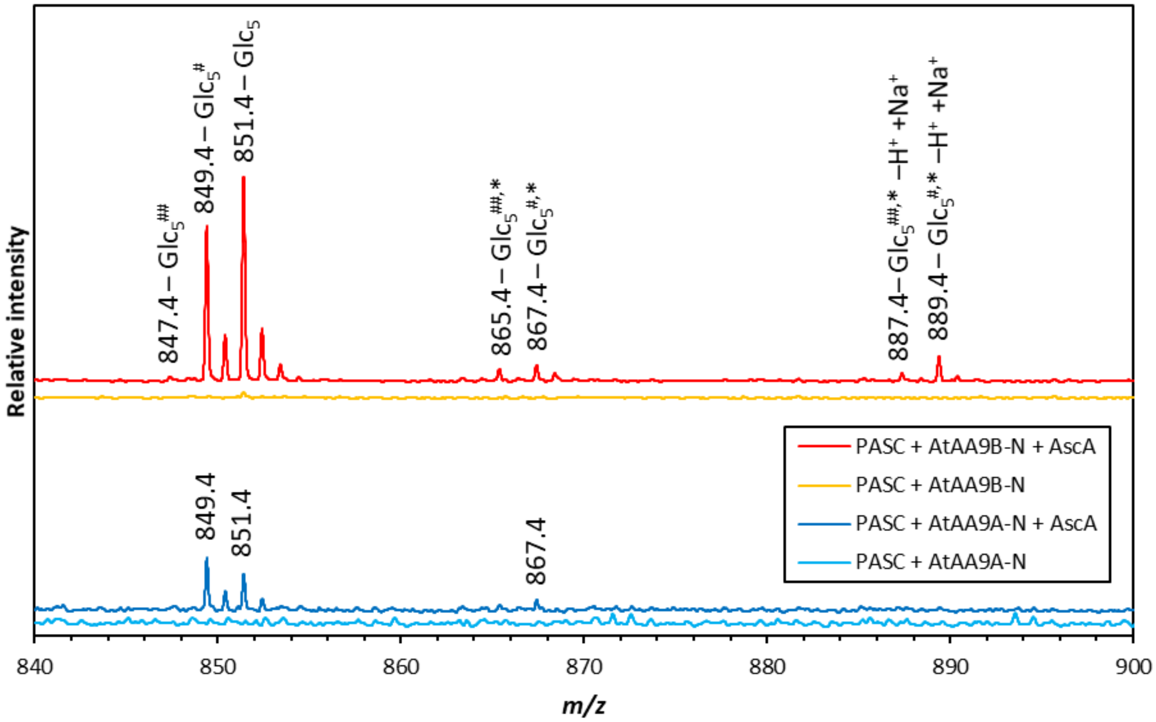

S4 Fig. MALDI-ToF spectra showing products generated upon incubation of *At*AA9A-N and *At*AA9B-N with PASC. The spectra show the DP 5 cluster displaying a pattern typical for C4-oxidation for *At*AA9A-N, while the pattern for *At*AA9B-N is typical for C1/C4-oxidation. Single or double oxidation is denoted with $^{\#}$ or $^{\#\#}$; hydration is denoted with *. The peaks at 887.4 and 889.4 correspond to the Na^+-salt of the aldonic acid, which is denoted as "$–H^+$ $+Na^+$". When it comes to regioselectivity, the clearest indicators are: absence of sodium salts of aldonic acids in the spectrum for *At*AA9A-N, which indicates the absence of C1-oxidation; presence of aldonic acids and double oxidized products in the spectrum for *At*AA9B-N, which indicates that both C1- and C4-oxidation occur. Further evidence for the regioselectivity of the two LPMOs is provided by the HPAEC chromatograms shown in Fig. 1 of the main manuscript.

```
                                        L2 loop
               10        20        30        40        50        60
McAA9B        1 HGYVSKAILDGKEYTGYLPYEDPYHNPP-PERIFRKI--AG---NGPI--EDLTSIDLQC 52
NcAA9M        1 HGFVDNATIGGQFY---QPYQDPYMGSP-PDRISRKI--PG---NGPV--EDVTSLAIQC 49
GtAA9A-2-N    1 HGYVDQVTIGGQVYTGYQPYQDPYE-SPVPQRIERAI--PG---NGPV--EDLTLLDIQC 52
GtAA9B        1 HGYVDTLNVGGTQYTGYLPYNDPYT-TPAPQRIERPI--PG---NGPV--TALTTIDVQC 52
FgAA9A-N      1 HGHVESITVGGTEYEGLNPGAAAFE-NPRKELAAWFATNTD---NGFVEPSAFGDADIIC 56
McAA9F        1 HGYVSSIQADGQTYPGADP----HNPNP--ESPGWQAENTD---LGFVEPSAFSTPAIAC 51
McAA9A-N      1 HGYVSGIVVDGAYHGGYIVDKYPYMPNP-PDVVGWSTTATD---LGFVAPDAFGDPDIIC 56
AtAA9B-N      1 HGYVSGAVVDGQYYSGYDMS-YHYMSDP-PKVIGWSTDATD---LGFVDGSSYADADIIC 55
TaAA9A        1 HGFVQNIVIDGKNYGGYLVNQYPYMSNP-PEVIAWSTTATD---LGFVDGTGYQTPDIIC 56
CvAA9A        1 HTRMFSVWVNGVDQGDGQ---N---------VYIRTP--PN---TDPI--KDLASPALAC 41
AtAA9A-N      1 HTTVHAVWINDVDQGEGN-SQS---------GYIRSP--PS---NSPI--TDVTSKDMTC 43
LsAA9A        1 HTLVWGVWVNGVDQGDGR---N---------IYIRSP--PN---NNPV--KNLTSPDMTC 41
McAA9H        1 HYTFPSLIANGVVTGEW--------------EYVRQT--ENHYSNAPV--TDVSSEAIRC 42
NcAA9A-N      1 HTIFVQLEADGTTYPVS--------------YGIRTP--SY---DGPI--TDVTSNDLAC 39
NcAA9D        1 HTIFSSLEVNGVNQGLG--------------EGVRVP--TY---NGPI--EDVTSASIAC 39
MYCTH_79765   1 HAIFQKVSVNGADQGSL--------------TGLRAP--NN---NNPV--QNVNSQDMIC 39
NcAA9C-N      1 HTIFQKVSVNGADQGQL--------------KGIRAP--AN---NNPV--TDVMSSDIIC 39
PaAA9H-N      1 HSIFQKVSVNGVDQGQL--------------KGVRAP--YS---NFPI--ENVNHPDFAC 39

Consensus       *  .     ..                                  :         . *

                  L2 loop                       L3 loop
                         70        80        90       100       110       120
McAA9B       53 GGWQNSGSAPAPLTAEPVTPGTVQKLQWT--------------TWPDSHKGPIITYMARC 98
NcAA9M       50 NADS----APAKLHASAAAGSTV-TLRWT--------------IWPDSHVGPVITYMARC 90
GtAA9A-2-N   53 NGSGGSGTKPAALIASAAAGDEI-AFHWT--------------TWPSSHVGPVITYMGKV 97
GtAA9B       53 NGENGGGSSPAPLVATIAAGGKI-AFHWT--------------TWPSSHVGPVITYLGKV 97
FgAA9A-N     57 HRGAE----NAVKSAKVKAGEKI-TIKWD--------------TWPESHKGPVIDYLASC 97
McAA9F       52 HKNAR----APPAHATVQAGSTI-KLTWN--------------TWPESHHGPVLDYIAPC 92
McAA9A-N     57 HRDGA----PGAIHAKVNAGATI-ELQWN--------------TWPESHHGPVIDYLANC 97
AtAA9B-N     56 HKNAK----NGAISAEIAAGKQV-ELQWT--------------DWPESHKGPVITYLANC 96
TaAA9A       57 HRGAK----PGALTAPVSPGGTV-ELQWT--------------PWPDSHHGPVINYLAPC 97
CvAA9A       42 NVKGG---EPVPQFVSASAGDKL-TFEWYRVK-------RGDDIIDPSHSGPITTWIAAF 90
AtAA9A-N     44 NVNN----KATAKTLEVKAGDKI-TFEWHHDSRS-----ESDDIIASSHNGPILVYMAPT 93
LsAA9A       42 NVDN----RVVPKSVPVNAGDTL-TFEWYHNT-------RDDDIIASSHHGPIAVYIAPA 89
McAA9H       43 YENPG---RPAAKTLSVAAGSTV-GFTVS--------------PSIY--HPGPLQFYMARV 83
NcAA9A-N     40 NGGPN-PTTPSDKIITVNAGSTV-KAIWRHTLTS-----GADDVMDASHKGPTLAYLKKV 92
NcAA9D       40 NGSPN-TVASTSKVITVQAGTNV-TAIWRYMLST--TGDSPADVMDSSHKGPTIAYLKKV 95
MYCTH_79765  40 GQ----SGSTSNTIIEVKAGDRI-GAWYQHVIGGAQFPNDPDNPIAKSHKGPVMAYLAKV 94
NcAA9C-N     40 NA----VTMKDSNVLTVPAGAKV-GHFWGHEIGGAAGPNDADNPIAASHKGPIMVYLAKV 94
PaAA9H-N     40 NTN---IQLRDNTVIKVPAGARV-GAWWGHEIGGAAGPNDPDHPIAASHKGPIQVYLAKV 95

Consensus                        .   :                     * **   ::
```

S5 Fig. (continued)

```
                                                  LS loop
                          130       140       150       160       170       180
                  ....|....|....|....|....|....|....|....|....|....|....|....|
McAA9B         99 P-G--DCSEYEP-GTDAVWFKIAEDGKH----DDG-SWASDPLIN-DV-PYEFTIPEGLA 147
NcAA9M         91 P-D-TGCQDWTPSASDKVWFKIKEGGREG---TSN-VWAATPLMTAP-ANYEYAIPSCLK 143
GtAA9A-2-N     98 P-SNTDITSYSPTGSDVIWFKIDEAGYE-----NG-KWAATDIMSAQNSTWTVTIPKALA 150
GtAA9B         98 P-SSTDVTKYSPTGSDVIWFKIDEGGYS-----NG-KWAATDVLSAQNSTWTVTIPSSLA 150
FgAA9A-N       98 G-SAG-CAKVDK--TSLKFFKIAEAGMTS----GG-KFASDDLIAAG-NTWEVTVPTSIK 147
McAA9F         93 N-GD--CSSASA--GSLNFVKIAEKGLISGS-NPG-FWAADELIQNG-NSWEVTIPANLA 144
McAA9A-N       98 N-GD--CSSVDK--TSLKFFKISEAGLNDGSNAPG-QWASDDLIANN-NSWTVTIPKSIA 150
AtAA9B-N       97 N-GD--CATVDK--TQLEFFKIDEKGLISG--SDN-TWASDNLISSN-NSWTVTIPSSIA 147
TaAA9A         98 N-GD--CSTVDK--TQLEFFKIAESGLINDDNPPG-IWASDNLIAAN-NSWTVTIPTTIA 150
CvAA9A         91 TS-----PTMDG--TGPVWSKIHEEGYDAST---K-SWAVDKLIANK-GMWDFTLPSQLK 138
AtAA9A-N       94 EK--------GT--AGNGWVKIAEDGYT--D---G-TWAVETLIKNR-GKHSVTVPD-VA 135
LsAA9A         90 AS--------NG--QGNVWVKLFEDAYNVTN---S-TWAVDRLITAH-GQHSVVVPH-VA 133
McAA9H         84 PDGQ-TADSWDG--SGQVWFKIFEQGPQIDP-SGL-TWPSDGL-----SQVQVTIPSSLP 133
NcAA9A-N       93 D-D---ALTDTG--IGGGWFKIQEDGYNN-----G-QWGTSTVITNG-GFQYIDIPACIP 139
NcAA9D         96 D-N---AATASG--VGNGWFKIQQDGMDS----SG-VWGTERVINGK-GRHSIKIPECIA 143
MYCTH_79765    95 D-N---AATASK--TGLKWFKIWEDTFNP----STKTWGVDNLINNN-GWVYFNLPQCIA 143
NcAA9C-N       95 D-N---AATTGT--SGLKWFKVAEAGLSN-----G-KWAVDDLIANN-GWSYFDMPTCIA 141
PaAA9H-N       96 N-N---AANAGT--SGLQWFKVAEQGLNN-----G-VWAVDNMISNG-GWHYFDMPSCVA 142

Consensus                           : *: :             :    :           :*  :

                               ▼▼                ▼                LC loop
                          190       200       210       220       230       240
                  ....|....|....|....|....|....|....|....|....|....|....|....|
McAA9B        148 PGNYIVRHELWALHAAWT------YPGAQVYPSCFQVKVVGDG-TQ-QPT-N--LVAFPG 196
NcAA9M        144 PGYYLVRHEIIALHSAYS------YPGAQFYPGCHQLQVTGSG-TK-TPS-S-GLVSFPG 193
GtAA9A-2-N    151 PGQYIVRHEIIALHQAET------YPGAQFYPDCFQVQVTGPG-TE-TPT-SQALVSFPG 201
GtAA9B        151 PGQYIVRHEIIALHQAQT------YPGAQSYPDCFQIRVTGSG-NK-TPS-GSYLVSFPG 201
FgAA9A-N      148 AGNYVLRHEIIALHAAGQ------ENGAQNYPQCFNLEVESDG-TA-EPA----GVAGTS 195
McAA9F        145 PGKYVLRHEIIALHSAGN------PNGAQAYPQCINLEVTGGG-SA-TPS----GQPATS 192
McAA9A-N      151 PGNYVLRHEIIALHSAGN------QNGAQNYPQCFNLEITSNG-SD-NPE----GVLGTE 198
AtAA9B-N      148 AGNYVMRHEIIALHSAGN------KDGAQNYPQCLNFKVTGGG-SD-KPE----GTLGTA 195
TaAA9A        151 PGNYVLRHEIIALHSAQN------QDGAQNYPQCINLQVTGGG-SD-NPA----GTLGTA 198
CvAA9A        139 PGKYMLRQEIVAHHESDATFDKNPKRGAQFYPSCVQVDVKGVG-GD-AVPDQ--AFDFNK 194
AtAA9A-N      136 AGEYLFRPEIIALHEGNR------EGGAQFYMECVQVKVTSSG-SK-TLP-E--GVSIPG 184
LsAA9A        134 PGDYLFRAEIIALHEADSLYSQNPIRGAQFYISCAQITINSSD-DS-TPLPA--GVPFPG 189
McAA9H        134 SGDYLLRVEQIGLHSASS------VNGAQFYLSCAQLTVTGGG-NG-NPG-P--LVSFPG 182
NcAA9A-N      140 SGQYLLRAEMIALHAASS------TAGAQLYMECAQINIVGGTGGTALPS-T--TYSIPG 190
NcAA9D        144 PGQYLLRAEMIALHAASN------YPGAQFYMECAQLNVVGGT-GA-KTP-S--TVSFPG 192
MYCTH_79765   144 DGNYLLRVEVLALHSAYS------QGQAQFYQSCAQINVSGGG-SF-TPA-S--TVSFPG 192
NcAA9C-N      142 PGQYLMRAELIALHNAGS------QAGAQFYIGCAQINVTGGG-SA-SPS-N--TVSFPG 190
PaAA9H-N      143 PGHYLMRVELLALHSASV------RGAAQFYMECAQIEITGSG-TN-TGS-N--FVSFPG 191

Consensus          * *:.* *  . * .           ** *  * :. : .
```

S5 Fig. (continued)

```
                            ▼ ▼   LC loop
                         250       260       270       280       290       300
                    ....|....|....|....|....|....|....|....|....|....|....|....|
McAA9B         197 EYTPDTPGVVYDIYQN---------NEP-YPIPGPPVWTPA------------------- 227
NcAA9M         194 AYKSTDPGVTYDAYQA---------A-T-YTIPGPAVFTC-------------------- 222
GtAA9A-2-N     202 GYTPTTPGITFNVYSG--------SI-TSYPIPGPPVWTS-------------------- 232
GtAA9B         202 AYTATTPGIAFNVYTN---------F-TSYPIPGPAVWTGN------------------- 232
FgAA9A-N       196 LYTASEKGIVFDLYNN---------PT-SYPIPGPKMNIA-------------------- 225
McAA9F         193 FYSPNDPGILFNLYQS---------FD-SYPIPGPAVWSG-------------------- 222
McAA9A-N       199 LYKADDPGILFNIYQP---------MD-SYPIPGPALYTG-------------------- 228
AtAA9B-N       196 LYKDTDPGILVNIYQT---------LS-SYTIPGPALYSG-------------------- 225
TaAA9A         199 LYHDTDPGILINIYQK---------LS-SYIIPGPPLYTG-------------------- 228
CvAA9A         195 GYKYSDPGIAFDMYTD---------FD-SYPIPGPPVWDAQDEGCCFIDGVDTTSVKEVV 244
AtAA9A-N       185 AYTATDKGILFNIYDS---------FD-SYPIPGPAVWDG-------------------- 214
LsAA9A         190 AYTDSTPGIQFNIYTT------P--AT-SYVAPPPSVWSGALGGSIAQ--VGDASL---- 234
McAA9H         183 AYSPTDPGLLINIYWP------I--PT-SYELPGPPVWRG-------------------- 213
NcAA9A-N       191 IYKATDPGLLVNIYS-------MSPSS-TYTIPGPAKFTC-------------------- 222
NcAA9D         193 AYSGSDPGVKISIYWP------P--VT-SYTVPGPSVFTC-------------------- 223
MYCTH_79765    193 AYSASDPGILINIYGATGQPDNN--GQ-PYTAPGPAPISC-------------------- 229
NcAA9C-N       191 AYSASDPGILINIYGGSGKTDNG--GK-PYQIPGPALFTC-------------------- 227
PaAA9H-N       192 AYTADHPGILVSIYDLQGRPTNG--GR-PYTIPGPAPLTC-------------------- 228

Consensus           *     *:  . *               *  * *

                         310
                    ....|....|
McAA9B             --------
NcAA9M             --------
GtAA9A-2-N         --------
GtAA9B             --------
FgAA9A-N           --------
McAA9F             --------
McAA9A-N           --------
AtAA9B-N           --------
TaAA9A             --------
CvAA9A         245 KQIICVLK 252
AtAA9A-N           --------
LsAA9A         235 -------E 235
McAA9H             --------
NcAA9A-N           --------
NcAA9D             --------
MYCTH_79765        --------
NcAA9C-N           --------
PaAA9H-N           --------

Consensus
```

S5 Fig. Multiple sequence alignment of the catalytic domains of AA9 LPMOs for which activity on xyloglucan has been demonstrated. The alignment was generated using T-Coffee's Expresso tool (http://tcoffee.crg.cat/apps/tcoffee/do:expresso) and reflects a structure-based alignment of crystal structures and three-dimensional models. Substitution-intolerant xyloglucan-active AA9s appear in red, substitution-tolerant xyloglucan-active AA9s appear in blue. Additional xyloglucan-active LPMOs with unknown cleavage specificity, which are not shown in this alignment, include MYCTH_85556 [23] and MYCTH_100518 [24] from *Myceliophthora thermophila*, *Tt*AA9E from *Thielavia terrestris* [28] and *Gc*AA9A and *Gc*AA9B from *Geotrichum candidum* [27]. The sequences of LPMOs that are not able to cleave soluble cello-oligosaccharides appear on a grey background. (Note that for *Nc*AA9M, activity on cello-oligosaccharides has not

been reported; its product profile on PASC (short cello-oligosaccharides with DP 2-4) is similar to that of *Nc*AA9C, indicating that activity on soluble cello-oligosaccharides is very likely [30].) The two histidines and the axial tyrosine that coordinate the active site copper are marked with blue triangles and are highlighted in blue. Additional residues potentially involved in substrate–protein interactions (as experimentally identified for *Nc*AA9C [31] and *Ls*AA9A [4, 20]) are highlighted in green and marked with green triangles. A few additional residues, which are discussed in the main text, because we speculate that they may affect xyloglucan binding or the ability to cleave water-soluble cello-oligosaccharides, appear in black frames.

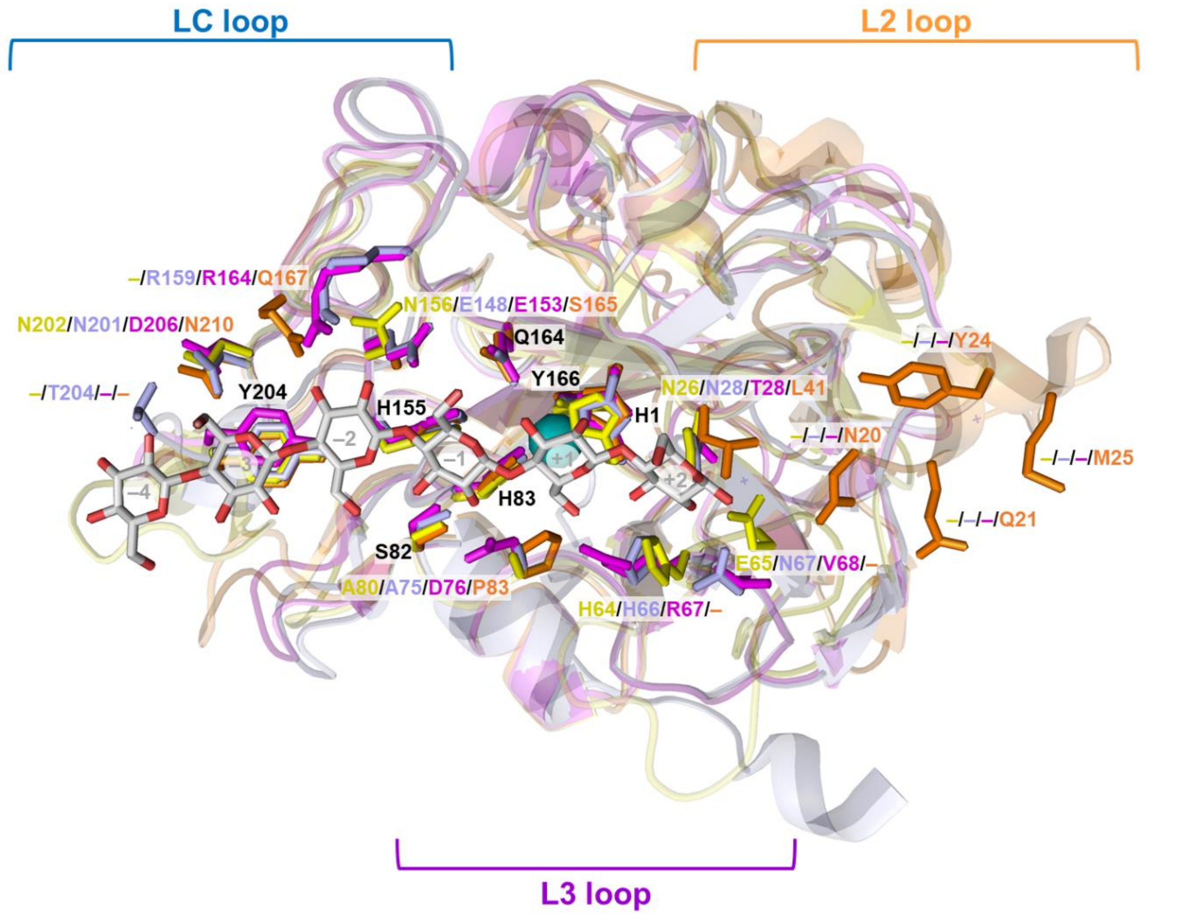

S6 Fig. Structural superposition of (substitution-intolerant) *Nc*AA9C-N (PDB: 4D7U; gold), *Ls*AA9A (PDB: 5ACI; light purple) and *Cv*AA9A (PDB: 5NLT; magenta) with (substitution-tolerant) *Ta*AA9A (PDB: 2YET; orange), showing surface-exposed side chains that (potentially) take part in protein–substrate interactions. The L2, L3 and LC loops are marked using the color coding used in S4 Fig. Side chain labels are colored according to the corresponding structure; the labels of fully conserved residues appear in black with numbering referring to *Nc*AA9C.

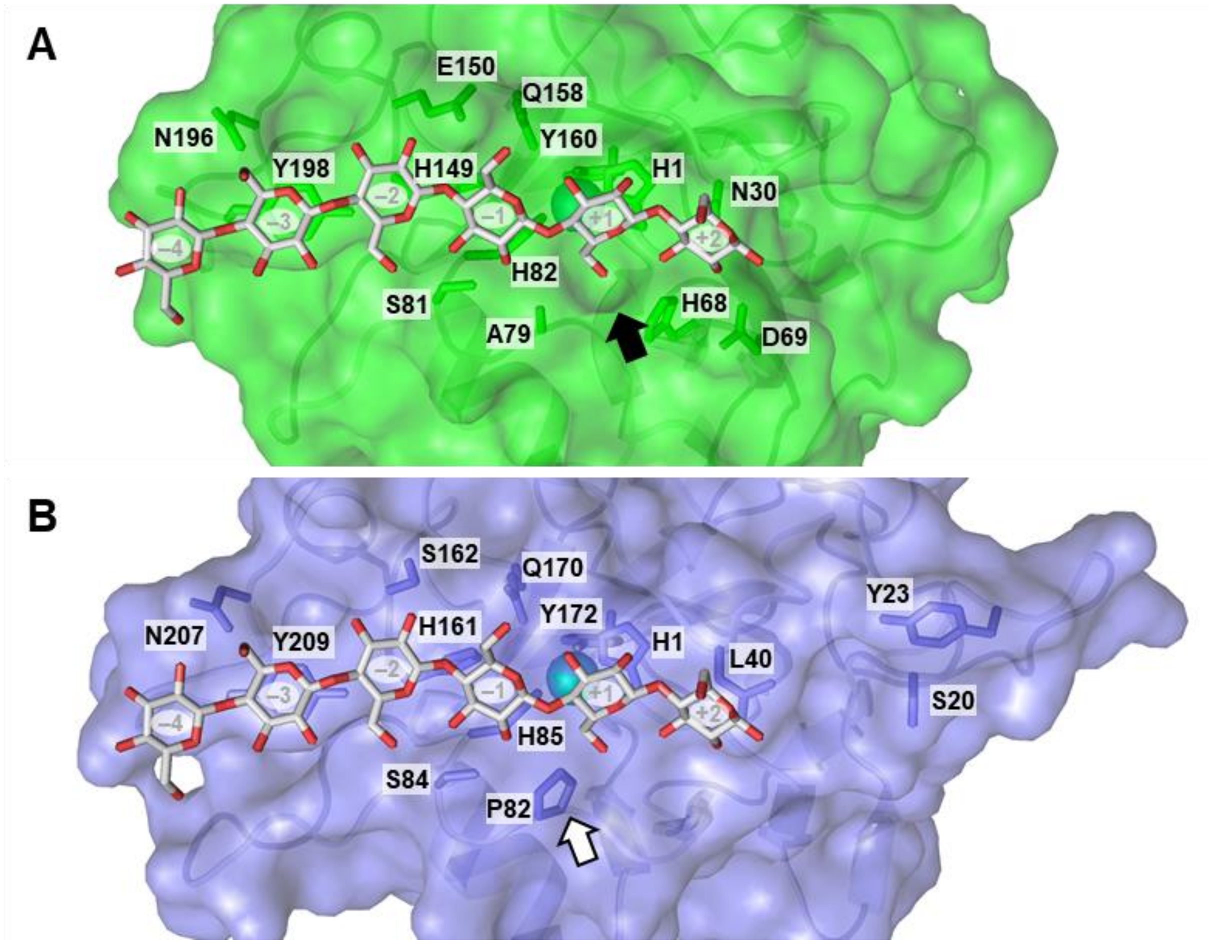

S7 Fig. The predicted substrate-binding surface of (A) *At*AA9A-N and (B) *At*AA9B-N. Structures were built with SWISS MODEL [32] based on the structures of *Ls*AA9A (PDB ID, 5N05) and *Ta*AA9A (PDB ID, 3ZUD), respectively. Analogously to Fig 7, panel **A** shows a cavity (black arrow) formed by the L3 loop of *At*AA9A, and panel **B** shows the lack of cavity and a conserved surface-exposed proline (white arrow) in *At*AA9B. The cellohexaose was superposed from the *Ls*AA9A–cellohexaose (PDB ID, 5ACI) structure.

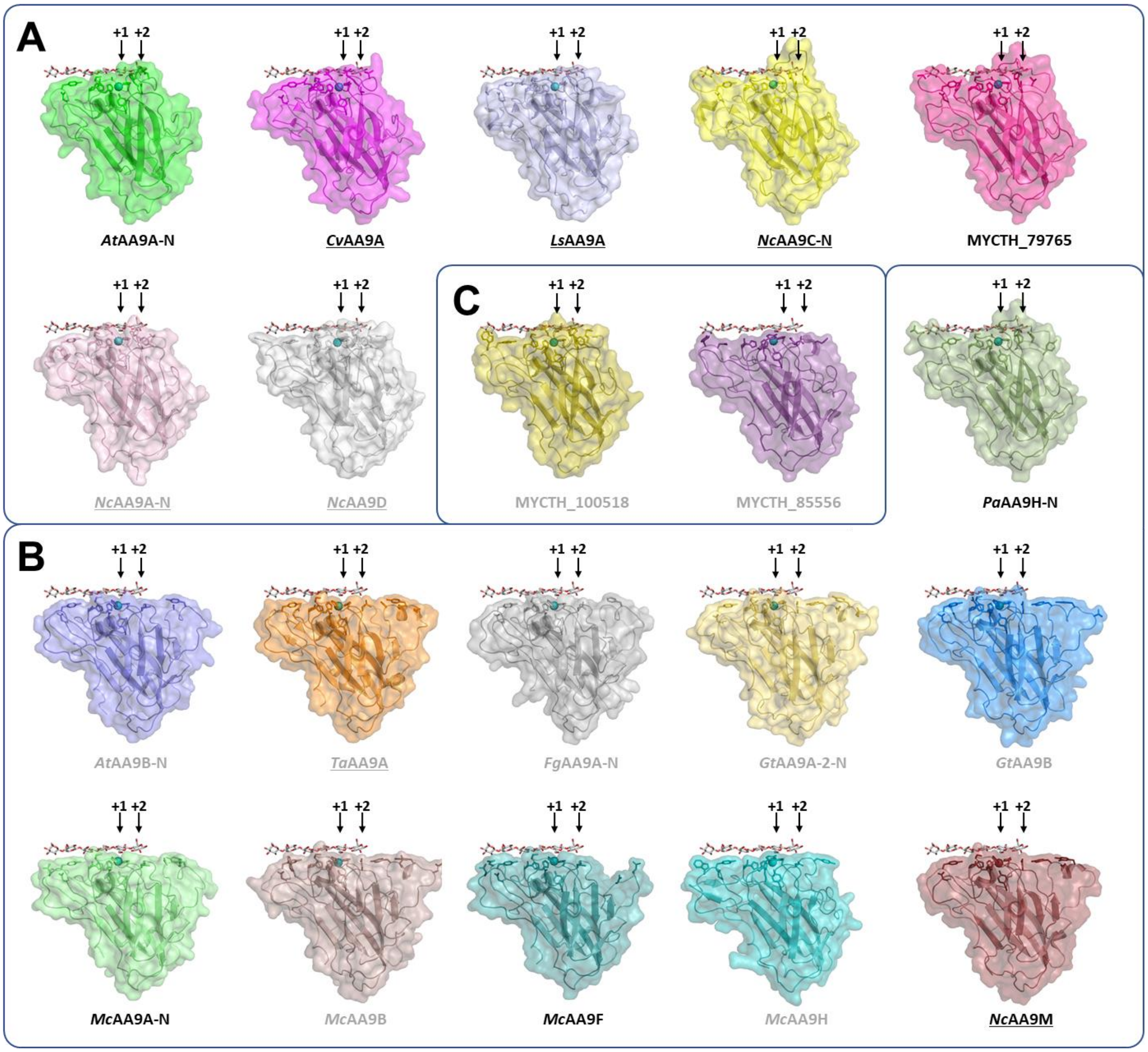

S8 Fig. Sideview of xyloglucan-active LPMOs which have (A) substitution-intolerant, (B) substitution-tolerant, or (C) unknown cleavage pattern. The names of LPMOs with known crystal structures (*Cv*AA9A, PDB: 5NLT; *Ls*AA9A, PDB: 5ACI; *Nc*AA9A-N, PDB: 5FOH; *Nc*AA9C-N, PDB: 4D7U; *Nc*AA9D, PDB: 4EIR; *Nc*AA9M, PDB: 4EIS; *Ta*AA9A, PDB: 2YET) are underlined. The other structures shown are models based on PDB structures with ID 3ZUD (*At*AA9B-N, *Fg*AA9A-N), 4B5Q (*Mc*AA9H), 4D7U (MYCTH_79765, *Pa*AA9H-N), 4EIR (MYCTH_100518), 4EIS (*Gt*AA9A-2-N, *Gt*AA9B, *Mc*AA9B), 4QI8 (MYCTH_85556), 5N05 (*At*AA9A-N) and 6H1Z (*Mc*AA9A-N, *Mc*AA9F). The positioning of cellohexaose ($Cell_6$) was modeled based on the *Ls*AA9A–$Cell_6$ structure (5ACI). Note that much of the variation between LPMOs occurs in the surface loops that make up the substrate-binding surface and that such loops are difficult to model accurately. Thus, the models cannot be used for detailed analysis of putative enzyme-substrate interactions.

The L3 loop appears as a protrusion of varying height behind the +1 and +2 subsites that are marked with an arrow and is present in all LPMOs in panel **A,** in *Pa*AA9H-N in panel **B,** and in MYCTH_100518 in panel **C**. The LPMOs in panel **B** (except *Pa*AA9H-N) and MYCTH_85556 in panel **C** have a more open and flat substrate-binding surface, which is extended towards the left (+ subsites) due to a longer L2 region (except in the case of *Mc*AA9H and *Pa*AA9H). The side chains of (putative) substrate-binding residues including the His-brace, surface-exposed aromatic residues and substrate-binding residues identified by NMR [31] and crystallography [20] are shown (see S4 Fig for more details). The names of LPMOs that are active on cello-oligosaccharides appear in black, whereas the names of LPMOs that are not active on cello-oligosaccharides appear in grey.

References

1. Midorikawa GEO, Correa CL, Noronha EF, Filho EXF, Togawa RC, Costa M, et al. Analysis of the transcriptome in *Aspergillus tamarii* during enzymatic degradation of sugarcane bagasse. Front Bioeng Biotechnol. 2018;6:123.
2. Kjaerbølling I, Vesth T, Frisvad JC, Nybo JL, Theobald S, Kildgaard S, et al. A comparative genomics study of 23 *Aspergillus* species from section *Flavi*. Nat Commun. 2020;11(1):1106.
3. Machida M, Asai K, Sano M, Tanaka T, Kumagai T, Terai G, et al. Genome sequencing and analysis of *Aspergillus oryzae*. Nature. 2005;438(7071):1157-61.
4. Frandsen KE, Simmons TJ, Dupree P, Poulsen JC, Hemsworth GR, Ciano L, et al. The molecular basis of polysaccharide cleavage by lytic polysaccharide monooxygenases. Nat Chem Biol. 2016;12(4):298-303.
5. Quinlan RJ, Sweeney MD, Lo Leggio L, Otten H, Poulsen JC, Johansen KS, et al. Insights into the oxidative degradation of cellulose by a copper metalloenzyme that exploits biomass components. Proc Natl Acad Sci USA. 2011;108(37):15079-84.
6. Petrović DM, Bissaro B, Chylenski P, Skaugen M, Sørlie M, Jensen MS, et al. Methylation of the N-terminal histidine protects a lytic polysaccharide monooxygenase from auto-oxidative inactivation. Protein Sci. 2018;27(9):1636-50.
7. Karlsson J, Saloheimo M, Siika-aho M, Tenkanen M, Penttila M, Tjerneld F. Homologous expression and characterization of Cel61A (EG IV) of *Trichoderma reesei*. Eur J Biochem. 2001;268(24):6498-507.
8. Pierce BC, Agger JW, Wichmann J, Meyer AS. Oxidative cleavage and hydrolytic boosting of cellulose in soybean spent flakes by *Trichoderma reesei* Cel61A lytic polysaccharide monooxygenase. Enzyme Microb Technol. 2017;98:58-66.
9. Hansson H, Karkehabadi S, Mikkelsen N, Douglas NR, Kim S, Lam A, et al. High-resolution structure of a lytic polysaccharide monooxygenase from *Hypocrea jecorina* reveals a predicted linker as an integral part of the catalytic domain. J Biol Chem. 2017;292(46):19099-109.
10. Jagadeeswaran G, Gainey L, Mort AJ. An AA9-LPMO containing a CBM1 domain in *Aspergillus nidulans* is active on cellulose and cleaves cello-oligosaccharides. AMB Express. 2018;8(1):171.
11. Pierce BC, Agger JW, Zhang Z, Wichmann J, Meyer AS. A comparative study on the activity of fungal lytic polysaccharide monooxygenases for the depolymerization of cellulose in soybean spent flakes. Carbohydr Res. 2017;449:85-94.
12. Jagadeeswaran G, Gainey L, Prade R, Mort AJ. A family of AA9 lytic polysaccharide monooxygenases in *Aspergillus nidulans* is differentially regulated by multiple substrates and at least one is active on cellulose and xyloglucan. Appl Microbiol Biotechnol. 2016;100(10):4535-47.
13. de Gouvêa PF, Gerolamo LE, Bernardi AV, Pereira LMS, Uyemura SA, Dinamarco TM. Lytic polysaccharide monooxygenase from *Aspergillus fumigatus* can improve enzymatic cocktail activity during sugarcane bagasse hydrolysis. Protein Pept Lett. 2019;26(5):377-85.
14. Lo Leggio L, Weihe CD, Poulsen JN, Sweeney M, Rasmussen F, Lin J, et al. Structure of a lytic polysaccharide monooxygenase from *Aspergillus fumigatus* and an engineered thermostable variant. Carbohydr Res. 2018;469:55-9.

15. Agger JW, Isaksen T, Várnai A, Vidal-Melgosa S, Willats WGT, Ludwig R, et al. Discovery of LPMO activity on hemicelluloses shows the importance of oxidative processes in plant cell wall degradation. Proc Natl Acad Sci USA. 2014;111(17):6287-92.
16. Nekiunaite L, Petrović DM, Westereng B, Vaaje-Kolstad G, Hachem MA, Várnai A, et al. *Fg*LPMO9A from *Fusarium graminearum* cleaves xyloglucan independently of the backbone substitution pattern. FEBS Lett. 2016;590(19):3346-56.
17. Kojima Y, Varnai A, Ishida T, Sunagawa N, Petrović DM, Igarashi K, et al. Characterization of an LPMO from the brown-rot fungus *Gloeophyllum trabeum* with broad xyloglucan specificity, and its action on cellulose-xyloglucan complexes. Appl Environ Microbiol. 2016;82(22):6557-72.
18. Kadowaki MAS, Várnai A, Jameson JK, AE TL, Costa-Filho AJ, Kumagai PS, et al. Functional characterization of a lytic polysaccharide monooxygenase from the thermophilic fungus *Myceliophthora thermophila*. PLoS ONE. 2018;13(8):e0202148.
19. Hegnar OA, Petrović DM, Bissaro B, Alfredsen G, Várnai A, Eijsink VGH. pH-dependent relationship between catalytic activity and hydrogen peroxide production shown via characterization of a lytic polysaccharide monooxygenase from *Gloeophyllum trabeum*. Appl Environ Microbiol. 2019;85(5):pii: e02612-18.
20. Simmons TJ, Frandsen KEH, Ciano L, Tryfona T, Lenfant N, Poulsen JC, et al. Structural and electronic determinants of lytic polysaccharide monooxygenase reactivity on polysaccharide substrates. Nat Commun. 2017;8(1):1064.
21. Bennati-Granier C, Garajova S, Champion C, Grisel S, Haon M, Zhou S, et al. Substrate specificity and regioselectivity of fungal AA9 lytic polysaccharide monooxygenases secreted by *Podospora anserina*. Biotechnol Biofuels. 2015;8:90.
22. Fanuel M, Garajova S, Ropartz D, McGregor N, Brumer H, Rogniaux H, et al. The *Podospora anserina* lytic polysaccharide monooxygenase *Pa*LPMO9H catalyzes oxidative cleavage of diverse plant cell wall matrix glycans. Biotechnol Biofuels. 2017;10:63.
23. Frommhagen M, Sforza S, Westphal AH, Visser J, Hinz SW, Koetsier MJ, et al. Discovery of the combined oxidative cleavage of plant xylan and cellulose by a new fungal polysaccharide monooxygenase. Biotechnol Biofuels. 2015;8:101.
24. Frommhagen M, Koetsier MJ, Westphal AH, Visser J, Hinz SW, Vincken JP, et al. Lytic polysaccharide monooxygenases from *Myceliophthora thermophila* C1 differ in substrate preference and reducing agent specificity. Biotechnol Biofuels. 2016;9(1):186.
25. Petrović DM, Várnai A, Dimarogona M, Mathiesen G, Sandgren M, Westereng B, et al. Comparison of three seemingly similar lytic polysaccharide monooxygenases from *Neurospora crassa* suggests different roles in plant biomass degradation. J Biol Chem. 2019;294(41):15068-81.
26. Hüttner S, Várnai A, Petrović DM, Bach CX, Kim Anh DT, Thanh VN, et al. Specific xylan activity revealed for AA9 lytic polysaccharide monooxygenases of the thermophilic fungus *Malbranchea cinnamomea* by functional characterization. Appl Environ Microbiol. 2019;85(23):pii: e01408-19.
27. Ladevèze S, Haon M, Villares A, Cathala B, Grisel S, Herpoël-Gimbert I, et al. The yeast *Geotrichum candidum* encodes functional lytic polysaccharide monooxygenases. Biotechnol Biofuels. 2017;10(1):215.
28. Cannella D, Mollers KB, Frigaard NU, Jensen PE, Bjerrum MJ, Johansen KS, et al. Light-driven oxidation of polysaccharides by photosynthetic pigments and a metalloenzyme. Nat Commun. 2016;7:11134.

29. Sun P, Laurent CVFP, Scheiblbrandner S, Frommhagen M, Kouzounis D, Sanders MG, et al. Configuration of active site segments in lytic polysaccharide monooxygenases steers oxidative xyloglucan degradation. Biotechnol Biofuels. 2020;13:95.
30. Vu VV, Beeson WT, Phillips CM, Cate JHD, Marletta MA. Determinants of regioselective hydroxylation in the fungal polysaccharide monooxygenases. J Am Chem Soc. 2014;136(2):562-5.
31. Courtade G, Wimmer R, Røhr ÅK, Preims M, Felice AK, Dimarogona M, et al. Interactions of a fungal lytic polysaccharide monooxygenase with beta-glucan substrates and cellobiose dehydrogenase. Proc Natl Acad Sci USA. 2016;113(21):5922-7.
32. Waterhouse A, Bertoni M, Bienert S, Studer G, Tauriello G, Gumienny R, et al. SWISS-MODEL: homology modelling of protein structures and complexes. Nucleic Acids Res. 2018;46(W1):W296-303.
